# Targeting the HSPA8‐CMA‐ATP6V1A Axis Triggers Lysosomal Hyperacidification and Catastrophic Vacuolation in Prostate Cancer

**DOI:** 10.1002/advs.76165

**Published:** 2026-06-19

**Authors:** Bingzheng An, Ze Gao, Shuo Chen, Liwei Meng, Chen Zhang, Kefan Song, Haochen Cui, Lei Yan, Zhiqing Fang

**Affiliations:** ^1^ Department of Urology Qilu Hospital of Shandong University Jinan Shandong China; ^2^ Department of Geriatric Medicine Jinan Hospital Jinan Shandong China

**Keywords:** chaperone‐mediated autophagy, HSPA8, lysosomal membrane permeabilization, osmotic stress, V‐ATPase

## Abstract

Prostate cancer (PCa) ranks among the most common and deadly malignancies worldwide. The clinical treatment of advanced prostate cancer is particularly challenging due to acquired drug resistance. Autophagy and lysosome‐related pathways are key drivers of this resistance. Targeting the lysosome represents a potential therapeutic strategy for PCa. In this study, we identified Heat Shock Protein Family A Member 8 (HSPA8) as a critical functional node of Aloperine (ALO). ALO suppresses autophagic flux, disrupts lysosomal homeostasis, and induces lysosomal vacuolation in cancer cells by inhibiting the function of HSPA8, impairing chaperone‐mediated autophagy (CMA)‐mediated ATP6V1A degradation. The resulting pathological accumulation and enhanced V1‐V0 association of the V‐ATPase complex drive pronounced lysosomal hyperacidification and severe osmotic swelling. This biochemical and physical stress is associated with lysosomal membrane permeabilization (LMP) and downstream loss of lysosomal integrity. Furthermore, we reveal that ALO‐induced vacuolation triggers a compensatory upregulation of cholesterol biosynthesis to buffer membrane expansion; preemptively disrupting this adaptive response with the DHCR7 inhibitor AY9944 yields significant synergistic lethality. Collectively, our findings reveal the specific cytotoxic mechanism of ALO and demonstrate that pharmacological targeting of the HSPA8‐CMA‐ATP6V1A axis is a valuable strategy for inducing lethal lysosomal vacuolation in advanced PCa.

## Graphical abstract

Our experimental evidence supports a model in which ALO targets the HSPA8‐CMA‐ATP6V1A axis to induce lysosomal hyperacidification and initiate osmotic and lipidomic stress. These changes are associated with LMP and loss of lysosomal integrity in prostate cancer cells.

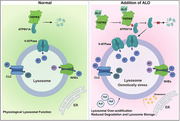

**Abbreviations**
ADT: Androgen Deprivation Therapy
ALO: Aloperine
ATP6V0D1: ATPase H^+^ Transporting V0 Subunit D1
ATP6V1A: ATPase H^+^ Transporting V1 Subunit A
Baf A1: Bafilomycin A1
CHX: Cycloheximide
CLCs: Cl^−^/H^+^ Exchangers
CMA: Chaperone‐mediated Autophagy
Co‐IP: Co‐immunoprecipitation
CQ: Chloroquine
CRPC: Castration‐resistant Prostate Cancer
DARTS: Drug Affinity‐responsive Target Stability
FBS: Fetal Bovine Serum
HSPA8: Heat Shock Protein family A Member 8
IF: Immunofluorescence
LAMP2A: Lysosomal‐associated Membrane Protein 2A
LMP: Lysosomal Membrane Permeabilization
MS: Mass Spectrometry
NHEs: Na^+^/H^+^ Exchangers
ORP: Oxysterol‐binding Protein‐related Protein
OSBP: Oxysterol‐binding Protein
PCa: Prostate Cancer
PI(4)P: Phosphatidylinositol 4‐phosphate
PI4K2A: Phosphatidylinositol 4‐kinase 2α
PS: Phosphatidylserine
SBD: Substrate‐binding Domain
SPR: Surface Plasmon Resonance
STR: Short Tandem Repeat
TEM: Transmission Electron Microscopy
V‐ATPase: Vacuolar‐type H^+^‐ATPase

## 1 Introduction

Prostate cancer (PCa) is one of the most prevalent malignancies and a leading cause of cancer‐related death among men in the world [1]. For patients with advanced or metastatic disease, androgen deprivation therapy (ADT) serves as the first‐line treatment [2]. Although initially effective, most patients eventually develop castration‐resistant prostate cancer (CRPC) with distant metastases, frequently involving bone [3, 4]. Preclinical studies have demonstrated that autophagy plays a dual role in both promoting disease progression and enhancing treatment resistance in patients with advanced PCa [5, 6]. Prostate cancer cells exploit and rely on heightened autophagy and other lysosomal processes to sustain survival and progression [7]. The classic lysosome/autophagy inhibitor chloroquine (CQ) has been evaluated in combination with agents such as enzalutamide in numerous preclinical studies and early‐phase clinical trials to overcome therapeutic resistance [8, 9]. Nevertheless, its features, including low specificity, moderate efficacy, and safety concerns, limit its clinical potential. Therefore, developing more effective and safer lysosome‐targeting drugs has become a shared priority in the field for treating prostate cancer.

Vacuolar‐type H^+^‐ATPase (V‐ATPase) is a highly conserved proton pump complex that plays a central role in maintaining pH homeostasis in multiple intracellular compartments and certain extracellular environments [10]. It serves not only as a proton pump for organelle acidification but also acts as a multifunctional hub that drives tumor progression, metabolic adaptation, and drug resistance [11, 12]. Disrupting the delicate turnover of V‐ATPase subunits and hijacking its proton‐pumping machinery has thus emerged as a potential new direction in anticancer therapy.

In recent years, the induction of significant lysosomal stress, often accompanied by the prominent accumulation of cytoplasmic vacuoles, has gained increasing attention as a strategy to eradicate apoptosis‐resistant cancer cells [13, 14, 15]. However, the physical expansion of vacuoles alone is frequently insufficient to execute terminal cell death [16, 17]. Mechanistically, it often results from severe disruption of intracellular homeostasis, triggered notably by lysosomal dysfunction [18, 19]. When excessive stress surpasses the elastic capacity of the lysosomal lipid bilayer, it triggers irreversible Lysosomal Membrane Permeabilization (LMP). LMP releases lethal luminal hydrolases into the cytosol, executing a definitive, non‐apoptotic death program. In recent years, an increased number of small‐molecule compounds have been reported to induce cytoplasmic vacuolation [15, 20, 21], some of which have been classified as methuosis or paraptosis. Nevertheless, the cellular origins of these vacuoles continue to be poorly defined. Therefore, identifying agents capable of driving lysosomal stress toward terminal LMP is expected to open new avenues for overcoming CRPC drug resistance.

In previous research, Aloperine (ALO), a quinolizidine alkaloid derived from the seeds and leaves of *Sophora alopecuroides*, was found to inhibit cancer cell proliferation and induce non‐apoptotic cell death in the preliminary screening [22, 23, 24, 25]. To explore the underlying mechanisms and potential targets of ALO, we continued the research and found that ALO targets HSPA8, disrupts vacuolar‐type H+‐ATPase (V‐ATPase) subunit ATP6V1A degradation. This specific blockade is associated with enhanced V1‐V0 association, lysosomal hyperacidification, and osmotic swelling, which can progress to LMP in prostate cancer cells. Furthermore, we uncover a metabolic vulnerability, demonstrating that targeting compensatory cholesterol biosynthesis synergistically sensitizes PCa cells to ALO‐induced osmotic stress. In summary, we characterize ALO as a specific lysosomal disruptor and establish the HSPA8‐CMA‐ATP6V1A axis as a viable therapeutic target for advanced prostate cancer.

## 2 Methods

### 2.1 Cell Culture

LNCaP and 22Rv1 cells were purchased from the Cell Bank/Stem Cell Bank, Chinese Academy of Sciences, and were cultured in 1640 medium (Gibco, Shanghai, China) supplemented with 10% fetal bovine serum (FBS) and 1% penicillin/streptomycin (MCE, USA). All the cells were cultured in a humid environment containing 5% carbon dioxide (BB150; Thermo Scientific, Shanghai, China) at 37°C. Cell line validation was performed using short tandem repeat (STR) analysis. Cells from passages 5–15 were used in this study to reduce genetic drift.

### 2.2 Chemicals and Reagents

Aloperine (Cat# A672020), AR7 (Cat# A857996), dynasore (Cat# A209594), EIPA (Cat# A367526), Ferrostatin‐1 (Cat# A228648), 3‐methyladenine (A145872), Z‐VAD (OMe) ‐FMK (Cat# A354225), necrostatin‐1 (Cat# A181851), NPPB (Cat# A336852), Monensin (Cat# A491565), Nigericin (Cat# A744611), and sorbitol (Cat# A256763) were purchased from Ambeed (Chicago, USA). CQ (Cat# Y264393), MG132 (Cat# Y210207), CHX (Cat# Y237498), RIPA lysis buffer (Cat# P0013B), proteinase inhibitor (Cat# P1005), and phosphatase inhibitor (Cat# P1045) were purchased from Beyotime (Shanghai, China). AY9944 (Cat# HY‐107420) and cholesterol (water soluble) (Cat# HY‐N0322A) were purchased from MedChemExpress (MCE, USA). Bafilomycin A1 (Cat# S1413) was purchased from Selleck Chemicals (Houston, USA).

### 2.3 Antibody

Antibodies for LAMP1 (Cat# 15665 or Cat# 9091), LAMP2A (Cat# 81197), Pan‐Cadherin (Cat# 4073T) and LC3B (Cat# 81197) were purchased from Cell Signaling Technology; antibodies for RAB5 (Cat# R25522), RAB7 (Cat# R25524) and ATP6V0D1 (Cat# R23564) were purchased from Zen BioScience (Chengdu, China); antibodies for PDZD8 (Cat# 25512‐1‐AP), SQSTM1 (Cat# 18420‐1‐AP), Beta Actin (Cat# 66009‐1‐Ig), ATP6V1A (Cat# 17115‐1‐AP), PI4K2A (Cat# 15318‐1‐AP), ATP6V0D1 (Cat# 68506‐1‐Ig), GAPDH (Cat# 10494‐1‐AP), HRP‐conjugated Goat Anti‐Rabbit IgG(H+L) (Cat# SA00001‐2), HRP‐conjugated Goat Anti‐Mouse IgG(H+L) (Cat# SA00001‐1), DYKDDDDK tag (Cat# 66008‐4‐Ig) and HA tag (Cat# 51064‐2‐AP) were purchased from Proteintech (Wuhan, China); ORP1 (Cat# 79H28K20)and ORP9 (Cat# 11E43E41) were purchased from Epizyme Biomedical Technology (Shanghai, China); HSPA8 (Cat# NB120‐2788) was purchased from Novus Biologicals (Centennial, USA); Galectin‐3 (Cat# YM4819) and Cathepsin D (Cat# YM8115) were purchased from Immunoway (JiangSu, China); AF488‐labeled Goat Anti‐Mouse IgG (H+L) (Cat# A0453) and AF555‐labeled Donkey Anti‐Rabbit IgG (H+L) (Cat# A0428) were purchased from Beyotime (Shanghai China).

### 2.4 Plasmid Construction

The human HSPA8 and ATP6V1A cDNA sequences were cloned into separate plasmid vectors for expression. Specifically, these constructions were generated by sub‐cloning or PCR‐based site‐directed mutagenesis and were inserted into modified pcDNA3.1 vectors containing N‐terminal HA or FLAG tags. All related plasmids are available from the corresponding author. To design microRNA‐based shRNA sequences for constructing gene‑specific lentiviral shRNA vectors, we utilized the SplashRNA online tool (http://splashrna.mskcc.org/). The resulting shRNA sequences, each encoded as a 97‑mer oligonucleotide, are listed in Table S2.

### 2.5 Cell Viability

Cell viability was assessed using CCK‐8 (K1018; Apex BIO, USA) and colony formation assays. For the CCK‐8 assay, cells were seeded at 1000 cells per well in 96‐well plates. To enable accurate time‐course tracking, independent parallel plates were established for each designated time point. Over the 6‐day treatment period, media containing ALO or vehicle control were freshly replaced every 24 h to maintain consistent drug exposure. At each 24‐h interval, 10 µL of CCK‐8 reagent was added to each well of the corresponding plate, followed by incubation for 2 h at 37°C. The absorbance was measured at 450 nm every 24 h over a 6‐day period. For the colony formation assay, cells were plated in 6‐well plates at a density of 1000 cells per well and cultured for 2 weeks, and the medium was replaced every 4 days. The resulting colonies were fixed with 4% paraformaldehyde (Servicebio, China) and stained with 0.1% crystal violet for visualization and quantification.

### 2.6 Live‐Cell Imaging

Live‐cell staining was performed using the following probes: ER‐Tracker Blue–White DPX (Yeasen, #40761ES50), LysoTracker Red DND‐99 (Yeasen, #40739ES50), LysoSensor Green DND‐189 (Yeasen, #40767ES50), MitoTracker Red CMXRos (Yeasen, #40741ES50), Lucifer Yellow (Ambeed, #A1158437), and FITC‐Dextran (Ambeed, #A1149843). Cells (5,000/dish) were treated with ALO for 24 h in 35 mm confocal dishes and then incubated with probes as indicated. Imaging was performed with an Olympus SpinSR10 confocal microscope.

### 2.7 Immunofluorescence (IF)

Cells were seeded in 35 mm confocal dishes at 5000 cells per dish and treated for 24–48 h. Following treatment, the cells were fixed with 4% paraformaldehyde (Beyotime, P0099) for 20 min at room temperature, permeabilized with 0.1% Triton X‐100 (Beyotime, P0096) for 4 min, and blocked with 1× blocking buffer (Beyotime, P0260) for 1 h. The cells were then incubated with the primary antibody diluted in blocking buffer overnight at 4°C. After three PBS washes, the samples were incubated with secondary antibodies for 30–60 min at room temperature and then were washed again. The nuclei were counterstained with DAPI for 2 min when necessary. Finally, the samples were allowed to settle for at least 1 h before imaging with an Olympus SpinSR10 confocal microscope.

### 2.8 Transmission Electron Microscopy (TEM)

Cells were initially fixed with 2.5% glutaraldehyde for 30 min at room temperature. This was followed by an overnight at 4°C, post‐fixation with one percent osmium tetroxide, and dehydration through a series of graded ethanol solutions. Subsequently, the cells were embedded in Spurr's resin to facilitate examination via transmission electron microscopy. Staining was accomplished with uranyl acetate and lead citrate on ultrathin sections placed on copper grids. Imaging was conducted using a Hitachi H‐7650 microscope.

### 2.9 Immunoblotting

Following treatment, the cells were lysed in RIPA buffer supplemented with PMSF, protease, and phosphatase inhibitors. Subcutaneous tumor tissues were minced and homogenized in the same lysis buffer. The lysates were centrifuged, and the protein concentration of the supernatant was determined using a bicinchoninic acid (BCA) assay kit (Beyotime, P0010). Equal amounts of protein were separated by SDS–PAGE and transferred onto PVDF membranes (Sigma–Aldrich, IPVH00010). After blocking, the membranes were incubated with specific primary and secondary antibodies. The protein bands were visualized using a Tanon 4800 imaging system (Tanon, Shanghai, China).

### 2.10 RNA Isolation and qPCR

Total RNA was isolated utilizing an RNA‐Quick purification kit (YiShan Biotech, China) in accordance with the manufacturer's instructions. cDNA was subsequently produced from equivalent quantities of RNA with Hifair III first Strand cDNA Synthesis SuperMix for qPCR (Yeasen, China). Quantitative real‐time PCR (qPCR) was conducted using a CFX Connect Real‐Time PCR Detection System (Bio‐Rad, USA) to quantify the expression levels of target mRNAs. All gene‐specific primers were procured from GenePharma (Shanghai, China), with their sequences enumerated in Table S1.

### 2.11 Molecular Docking

Protein–Protein Docking: To explore the interaction interface between HSPA8 and its downstream substrate ATP6V1A, protein–protein docking was conducted. The 3D structural models of human HSPA8 (UniProt ID: P11142) and ATP6V1A (UniProt ID: P38606) were obtained from the UniProtKB database. Docking simulations were performed using the GRAMM web server with its default parameters. The top ten models, ranked by predicted binding affinity, were retained for further analysis. The highest‐ranked pose, representing the most thermodynamically favorable binding conformation, was selected based on its docking score and subsequently visualized using PyMOL. Small Molecule–Protein Docking: To predict the binding mode and affinity between the small molecule ALO and HSPA8, molecular docking was performed. The 3D structure of the ALO ligand was retrieved from the PubChem database (CID: 162147). Energy minimization of the ALO ligand was conducted, and the structure was converted into PDBQT format using AutoDockTools (version 1.5.6). The 3D structural model of human HSPA8 (UniProt ID: P11142) was utilized as the receptor. Prior to docking, the receptor protein was prepared in PyMOL by removing water molecules and heteroatoms. Subsequently, polar hydrogens and Gasteiger charges were assigned using AutoDockTools. Molecular docking simulations were executed using AutoDock Vina (version 1.1.2) with a grid box centered on the putative binding pocket. The conformation exhibiting the lowest binding energy (kcal/mol) was selected as the optimal binding pose for further visualization and structural analysis.

### 2.12 RNA‐seq and Enrichment Analysis

Cells were treated with ALO or PBS for 24 h. Total RNA was isolated and extracted using an RNA‐Quick purification kit (YiShan Biotech, China) according to the manufacturer's instructions, and the samples were then sent to Bohao Technology (Shanghai, China) for RNA sequencing. Here, genes with a fold change ≥ 1 and a *p* value < 0.05 were defined as differentially expressed. Enrichment analysis was conducted using the Sangerbox online platform (http://sangerbox.com/login.html).

### 2.13 SPR Assay

The Plex Array HTA100 system was used to measure how strongly the recombinant HSPA8 protein (MedChemExpress, Cat# HY‐P73915A) is bound to ALO. First, 1 µg of the recombinant HSPA8 protein was attached to an activated 3‐D photo‐crosslinked chip. Then, the chip was exposed to different concentrations of the drugs, flowing over the surface at a set rate, to determine the binding affinity.

### 2.14 Drug Affinity Responsive Target Stability (DARTS) Assay

Cell lysates were prepared using lysis buffer, and the protein concentration was determined as described earlier. The cell lysates were then mixed with either ALO or PBS (up to 400 µm) for 2.5 h at room temperature to allow for binding. After this, pronase (MCE, Cat# HY‐114158) was added, and the mixture was incubated for 30 min. The samples were then separated using SDS–PAGE, and the gel was stained with a PAGE Gel Silver Staining Kit (Yeasen, Cat# 36244ES25) to prepare for mass spectrometry identification.

### 2.15 Lysate‐Based CETSA / Thermal Shift Assay

The cells were resuspended in PBS supplemented with 1% PMSF and lysed through repeated freeze–thaw cycles within liquid nitrogen. The resultant lysate was then partitioned into two equal portions: one underwent ALO treatment, while the other received an equivalent volume of PBS as a control, and both were incubated at ambient temperature for a duration of 1 h. Each aliquot was subsequently subdivided and subjected to heating at varying temperatures (37, 43, 47, 53, 57, 63, and 67°C) for a period of 3 min. The stability of the proteins was then evaluated using Western blot analysis.

### 2.16 Nude Mouse Xenograft Model

BALB/c nude mice (four weeks old, Vital River, China) were maintained under standard conditions, with all procedures approved by the Animal Ethics Committee of Qilu Hospital, Shandong University. Subcutaneous xenografts were established by injecting 2×106 22Rv1 cells into the right flanks of the mice. After seven days of post‐inoculation, the mice were randomly divided into three groups (n = 6 per group): a vehicle control group (PBS), a low‐dose ALO group (10 mg/kg), and a high‐dose ALO group (50 mg/kg). Treatments were administered via intraperitoneal injection every other day. At the experimental endpoint, blood samples were collected from all mice for systemic safety evaluation; within each group, samples from three mice were allocated for complete blood count (CBC) analysis, while samples from the remaining three mice were subjected to serum biochemistry testing. Following blood collection, the mice were euthanized, and the tumors were excised, photographed, and weighed. Tumor volumes were calculated using the formula: V = (length × width^2^) × 0.52.

### 2.17 Generation of Stable Cell Lines

To establish cell lines with stable HSPA8‐Flag expression, lentiviruses carrying the HSPA8‐Flag construct were packaged by IGE Biotechnology (Guangzhou, China). LNCaP and 22Rv1 cell lines were plated in 48‐well plates at a density of 1 × 10^5^ cells per well. They were then infected with lentivirus (5 µL of 1 × 10^8^ TU/mL) and 2 µL of polybrene when they reached 20%–30% confluence. After 72 h, puromycin was added to the culture medium to select for cells that had been stably transduced, and the resistant clones were then expanded.

### 2.18 Coimmunoprecipitation Assay

Cell lysates were prepared using IP lysis buffer (P0013; Beyotime), followed by overnight incubation at 4°C with the appropriate antibody. Protein A/G MagBeads (IP Grade) (Cat# 36417ES03, Yeasen) were subsequently added for 4 h of incubation. After the samples were washed, Western blot analysis was performed.

### 2.19 Transfection

Cells at 60%–70% confluence were transfected using Lipofectamine 2000 Transfection Reagent (Yeasen, 40802ES01) according to the manufacturer's protocol. Briefly, plasmids or siRNAs were mixed with the transfection reagent in Opti‐MEM (Gibco, 31985070) to form complexes, which were then added to the cells. After 72 h of incubation, the cells were harvested for subsequent analysis. All the plasmid and siRNA sequences used are listed in Table S2.

### 2.20 Lysosomal pH Assay

Lysosome pH was detected using a Lysosomal Acidic pH Detection Kit (#L266, Dojindo, Japan). Cells (5000/sample) were sequentially stained with LysoPrime Green and pHLys Red for 30 min each, washed twice with PBS, and resuspended in 3% FBS/PBS. For standard curve generation, pH calibration buffers were prepared as previously described [26]. The stained cells were incubated in 100 µL of each pH titration buffer containing 10 µm nigericin and monensin at 37°C for 10 min. Fluorescence intensity was then recorded using a Tecan Infinite E plex microplate reader. Lysosomal pH was calculated using the pHLys Red/LysoPrime Green fluorescence intensity ratio based on the standard curve (Figure S6D).

### 2.21 Immunofluorescence Analysis of Galectin‐3

Cells were seeded in 35 mm confocal dishes at 5000 cells per dish and treated as indicated, washed with PBS, and fixed in 4% PFA for 15 min. After permeabilization with 0.1% Triton X‐100 for 10 min and blocking with 5% BSA for 1 h, cells were incubated with an anti‐Galectin‐3 primary antibody and an anti‐Lamp1 primary antibody, overnight at 4°C. Cells were then stained with a fluorescently labeled secondary antibody for 1 h at room temperature in the dark. The nuclei were counterstained with DAPI for 2 min when necessary. Finally, the samples were allowed to settle for at least 1 h before imaging with an Olympus SpinSR10 confocal microscope.

### 2.22 Evaluation of Chaperone‐Mediated Autophagy (CMA) Activity

LNCaP and 22Rv1 cells were generated to stably express PAmCherry‐KFERQ‐NE by lentiviral transduction. Stably transduced cells were seeded in 35 mm confocal dishes at 5000 cells per dish, cells were then photoactivated using a 405 nm laser to induce PAmCherry fluorescence. For the assay, cells were treated with 200 µm ALO, or 10 µm AR7 for 16 h. Images were immediately captured using an Olympus SpinSR10 confocal microscope. To precisely evaluate reporter clearance, the fluorescent puncta area per cell was quantified using ImageJ software.

### 2.23 Statistical Analysis

Data from all the experiments, which were conducted in duplicate and repeated at least three times independently, are expressed as the mean ± SD. Statistical significance was determined using GraphPad Prism 9.0 (GraphPad Software, CA, USA). The sample sizes are provided in the figure legends. Group differences were assessed by two‐tailed unpaired *t*‐tests (two groups) or one‐way ANOVA with post hoc tests (three or more groups) using GraphPad Prism 9. Significance was defined as *p* < 0.05 and denoted as follows: ns, not significant; ^*^ *p* < 0.05, ^**^ *p* < 0.01, ^***^ *p* < 0.001.

## 3 Results

### 3.1 ALO Inhibits Prostate Cancer Cell Growth and Induces Cytoplasmic Vacuolation In Vitro and In Vivo

**FIGURE 1:**
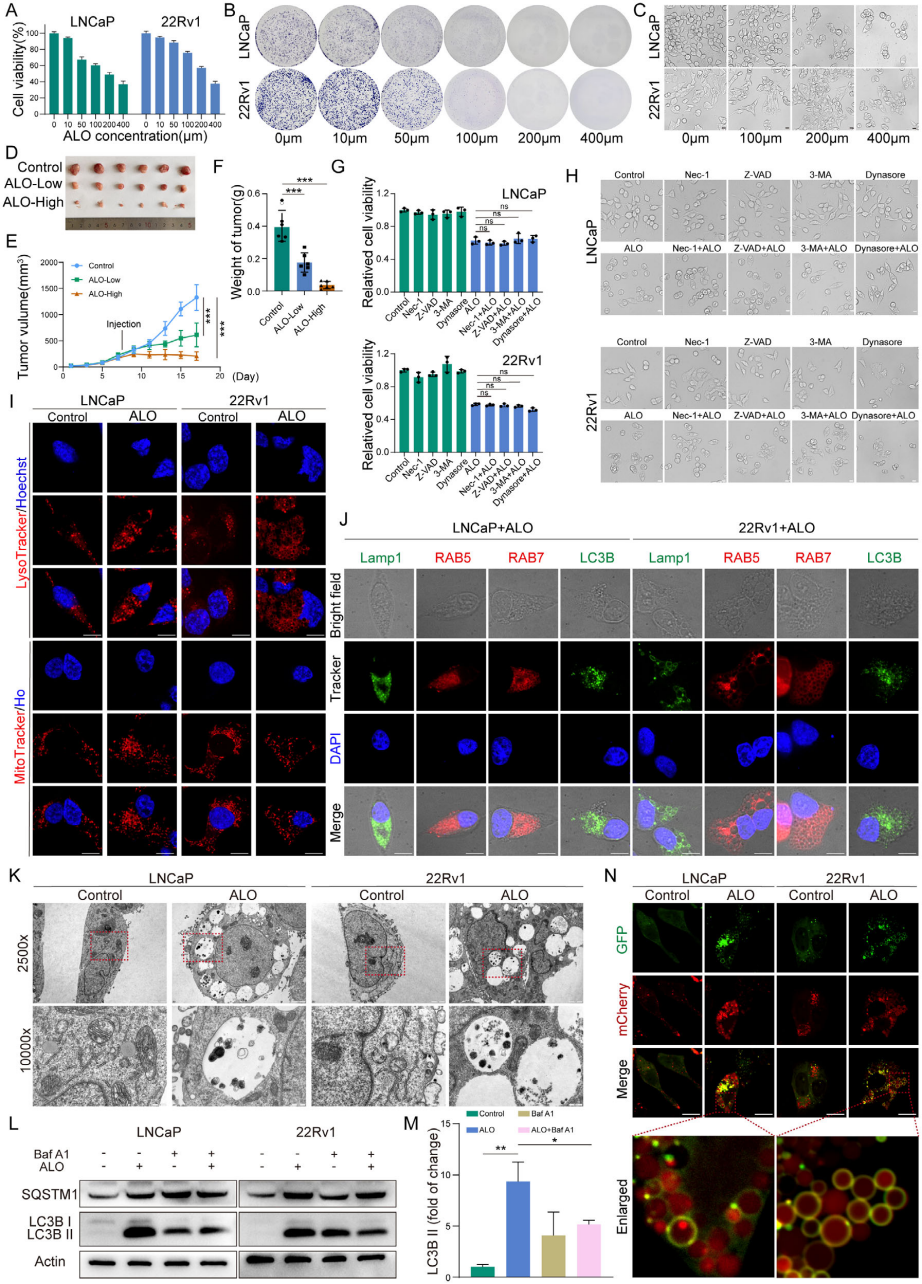
ALO inhibits prostate cancer cell proliferation and induces lysosomal vacuolation. (A) Viability of LNCaP and 22Rv1 cells treated with the indicated concentrations of ALO for 24 h, as assessed by a CCK‐8 assay (n = 3). (B) Representative images of colony formation assays with LNCaP and 22Rv1 cells (n = 3). (C) Bright field microscopy images showing cytoplasmic vacuolation in LNCaP and 22Rv1 cells after 24 h of ALO treatment. (D–F) In vivo antitumor efficacy of ALO in a 22Rv1 xenograft model: (D) representative tumor images, (E) tumor growth curves, and (F) excised tumor weights. (G) Cell viability and (H) bright field images demonstrating that ALO‐induced vacuolation and cell death do not occur by major cell death pathways. Cells were cotreated for 24 h with 200 µm ALO and inhibitors of apoptosis (Z‐VAD‐FMK, 50 µm), autophagy (3‐MA, 1 mm), necroptosis (necrostatin‐1, 50 µm), or methuosis (Dynasore, 30 µm). (I) Live‐cell imaging of LNCaP and 22Rv1 cells treated with 200 µm ALO for 24 h and stained with LysoTracker, MitoTracker, and Hoechst. (J) Immunofluorescence analysis of LC3B, Rab7, LAMP1, and Rab5 expression in cells treated with 200 µm ALO for 24 h. (K) Representative TEM images of LNCaP and 22Rv1 cells following 24 h of treatment with 200 µm ALO. (L, M) Immunoblot analysis (L) and quantification (M) of LC3B‐II and SQSTM1/p62 levels in cells treated with 200 µm ALO, 1 µm BafA1, or their combination for 24 h (n = 3). (N) Fluorescence images of LNCaP and 22Rv1 cells expressing the mCherry‐GFP‐LC3B reporter to monitor autophagic flux after ALO treatment. Data are presented as mean ± SD. ^*^ *p* < 0.05, ^**^ *p* < 0.01, ^***^ *p* < 0.001. Scale bar: 10 µm.

We first evaluated the cytotoxic and inhibitory effects of ALO on human LNCaP and 22Rv1 PCa cells. Our findings demonstrated that ALO suppressed the growth of these cells in a time‐ and dose‐dependent manner (Figure 1A and Figure S1A). We subsequently employed a colony formation assay to assess the sustained antiproliferative effect, which revealed a dose‐dependent suppression of colony formation in both cell lines (Figure 1B). Morphological assessments further revealed that ALO induced cytoplasmic vacuolation, with the extent and size of the vacuoles being concentration‐dependent (Figure 1C).

We then evaluated the cytotoxicity of ALO in the normal prostate epithelial cell line RWPE‐1. The CCK‐8 assay showed that RWPE‐1 cells were less sensitive to ALO, with a calculated IC50 of 740.5 µm at 48 h (Figure S1B). For comparison, we tested the conventional lysosomal inhibitor CQ. CQ treatment for 48 h reduced the viability of 22Rv1 cells with an IC50 of 44.54 µm (Figure S1C). However, CQ also reduced the viability of normal RWPE‐1 cells with a similar IC50 of 69.87 µm (Figure S1D). Colony formation assays confirmed that RWPE‐1 cells maintained their proliferation capacity at ALO concentrations up to 400 µm (Figure S1E). These data suggest preliminary in vitro selectivity of ALO between prostate cancer cells and normal epithelial cells relative to CQ under the tested conditions.

Time‐course morphological observation showed that ALO induced progressive vacuolation and subsequent cell death in 22Rv1 cells from 4 to 48 h. Under the same treatment conditions, RWPE‐1 cells exhibited similar lysosomal swelling or vacuolation (Figure S1F). Removing the drug after 24 h (wash‐out) failed to reverse the morphological changes or restore cell proliferation in 22Rv1 cells, indicating that the ALO‐induced cellular damage is irreversible (Figure S1F). However, the vacuolation in RWPE‐1 cells was substantially reversed when removing the drug after 24 h. These results suggest that ALO‐induced vacuolation is more reversible in RWPE‐1 cells, whereas 22Rv1 cells show persistent morphological damage and impaired proliferative recovery after washout.

Cytoplasmic vacuolation induced by ALO was also observed across a variety of other tumor cell lines (Figure S1G), suggesting that the induction of cytoplasmic vacuolation represents a generalized cellular response to ALO treatment.

To determine the antitumor effect of ALO in vivo, we established a 22Rv1 xenograft model in BALB/c nude mice. The mice were treated with vehicle control, a low dose of ALO (ALO‐Low), or a high dose of ALO (ALO‐High). Tumor‐bearing mice were randomized to receive either PBS or ALO via intraperitoneal injection every two days, starting seven days post‐inoculation. Compared with control (PBS) treatment, both low and high dose of ALO treatment significantly suppressed tumor growth, as evidenced by a significant decrease in final tumor volume and weight (Figure 1D–F). To assess the potential side effects of ALO, we monitored the mice during the in vivo experiments. Serum markers for liver and kidney function remained within normal ranges (Figure S2). Furthermore, histological examination of the lung, liver, spleen, kidney, and heart showed no signs of tissue damage compared to the control group (Figure S3A). ALO administration did not negatively affect mouse body weight (Figure S3B). Overall, no overt toxicity was detected under the tested conditions.

### 3.2 ALO‐Induced Vacuoles Originate From the Lysosomal/Late Endosomal Compartment

To elucidate the mechanism underlying ALO‐induced cell death and cytoplasmic vacuolation in PCa cells, a panel of inhibitors targeting key cell death pathways (including Z‐VAD‐FMK, 3‐methyladenine, dynasore, and necrostatin‐1) were applied to determine their ability to rescue cell viability. None of these inhibitors attenuated vacuolation or restored cell viability (Figure 1G,H), indicating that ALO‐induced cell death is independent of canonical apoptosis, autophagy, methuosis, or necroptosis.

We next sought to identify the origins of these vacuoles. Live‐cell imaging of 22Rv1 and LNCaP cells showed that ALO‐induced vacuoles were stained by LysoTracker but not by MitoTracker (Figure 1I). This specific colocalization strongly suggests that the vacuoles originate from lysosomes or endosomes.

Vacuole formation is a hallmark of micropinocytosis; therefore, our initial aim was to ascertain if the vacuoles generated by ALO were a consequence of micropinocytosis. To this end, we utilized fluid‐phase tracers. Neither Lucifer yellow nor FITC‐dextran demonstrated substantial colocalization with the vacuoles (Figure S3C,D), suggesting a mechanism distinct from those linked to micropinocytosis and methuosis. Furthermore, we investigated whether the ALO‐induced vacuoles were derived from the lysosome by using immunofluorescence targeting Rab7 (late endosomes), LAMP1 (lysosomes), Rab5 (early endosomes), and LC3 (autophagosomes). The vacuoles strongly co‐localize with the LAMP1 and RAB7 but weakly co‐localize with RAB5 and LC3 (Figure 1J and Figure S4A,B). This marker profile supports a model in which vacuoles form by the fusion of early endosomes and autophagosomes with swollen lysosomes. Ultrastructural analysis by TEM revealed that ALO‐induced vacuoles were single‐membrane‐bound and frequently contained undigested cytoplasmic content, which is consistent with a lysosomal/late endosomal identity (Figure 1K). These findings are highly consistent with those of previous reports on vacuolin‐1, a compound that selectively induces lysosome enlargement [27, 28], supporting the conclusion that these vacuoles are derived from the lysosomal/late endosomal compartment.

### 3.3 ALO Mediates LC3B‐II and SQSTM1 Accumulation

Despite TEM evidence excluding vacuoles as autophagosomes, their apparent labeling with EGFP‐LC3B raised the following question: could basal autophagy contribute membrane material to their expansion? Therefore, we assessed the status of the canonical autophagy markers microtubule‐associated protein 1 light chain 3B (LC3B) [29] and p62 [30]. Interestingly, immunoblotting revealed that ALO treatment precipitated a concomitant elevation of LC3B‐II and p62, signifying a substantial blockade of autophagic flux rather than mere induction of phagophore formation in LNCaP and 22Rv1 cells (Figure 1L,M).

ALO treatment induced a significant reorganization of the autophagic pathway, as revealed by the mCherry‐GFP‐LC3B reporter. Conventional yellow (autophagosomes) and red (autolysosomes) puncta were largely replaced by large vacuoles whose membranes appeared yellow and whose interiors emitted red fluorescence (Figure 1N). These unique morphological features indicate that ALO inhibits normal autophagic degradation and induces the aberrant accumulation and fusion of autophagosomal and lysosomal compartments, which is consistent with the findings of previous reports [19, 31, 32]. Based on these findings, we suggest that ALO might disrupt lysosomal breakdown processes, leading to a significant buildup of autolysosomes. These accumulated autophagic structures could then cause vacuolar expansion through processes like membrane fusion. This model provides a mechanistic perspective on how ALO causes vacuolation.

### 3.4 V‐ATPase Hyperassembly Drives Lysosomal Hyperacidification

**FIGURE 2:**
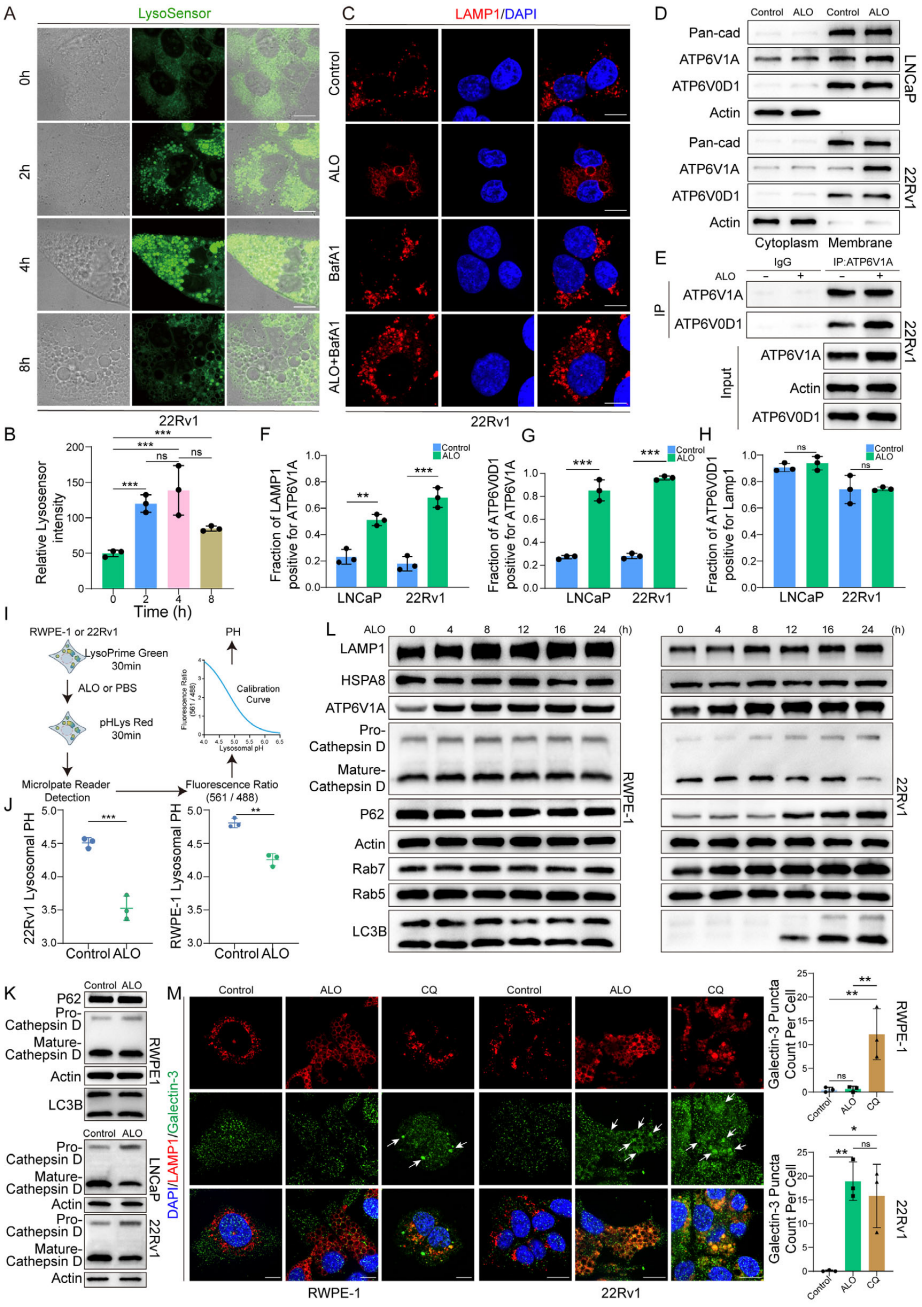
ALO triggers V‐ATPase hyperassembly and LMP. (A) Representative images and (B) quantification of LysoSensor Green fluorescence in 22Rv1 cells treated with PBS or 200 µm ALO, revealing enhanced lysosomal acidification (n = 3). (C) LAMP1 immunofluorescence images of 22Rv1 cells treated with 200 µm ALO, 1 µm BafA1, or their combination for 24 h, demonstrating that BafA1 rescues ALO‐induced vacuolation. (D) Immunoblot analysis of ATP6V1A and ATP6V0D1 expression in membrane fractions from LNCaP and 22Rv1 cells treated with 200 µm ALO or PBS, indicating enhanced V‐ATPase V1‐V0 domain assembly. (E) Co‐IP assay demonstrating enhanced interaction between ATP6V1A and ATP6V0D1 following ALO treatment. (F–H) Quantitative analysis of the colocalization fraction between LAMP1 and ATP6V1A (F) in Figure S6A, LAMP1 and ATP6V0D1 (G) in Figure S6B, and ATP6V1A and ATP6V0D1 (H) in Figure S6C upon ALO treatment. (I) Schematic illustration of the ratiometric Lysosomal Acidic pH Detection assay. (J) Absolute quantification of lysosomal pH in 22Rv1 and RWPE‐1 cells after ALO treatment. (K) Immunoblot analysis of Cathepsin D (CTSD), p62, and LC3B expression in LNCaP, 22Rv1, and normal RWPE‐1 cells treated with ALO. (L) Time‐course immunoblotting of CTSD, p62, and LC3B in RWPE‐1 and 22Rv1 cells treated with ALO for up to 24 h. (M) Representative immunofluorescence images and quantitative bar charts of LAMP1 (red) and Galectin‐3 (green) in RWPE‐1 and 22Rv1 cells. Cells were treated with vehicle, ALO, or CQ. The number of Galectin‐3 puncta per cell was quantified to evaluate lysosomal membrane permeabilization (LMP). Data are presented as mean ± SD. ^*^ *p* < 0.05, ^**^ *p* < 0.01, ^***^ *p* < 0.001. Scale bar: 10 µm.

Lysosomal vacuolation, characterized by the emergence of sizable vacuoles, is a feature of various pathophysiological conditions, including lysosomal storage disorders, aging, infectious diseases, and neurodegeneration [33, 34, 35]. Contrary to the initial hypothesis predicting lysosomal alkalization, ALO prompted the formation of numerous vacuoles that exhibited strong staining with LysoTracker, suggesting increased acidity. Next, Lysosensor Green was utilized to evaluate whether ALO affected lysosomal acidification. The increased fluorescence intensity of LysoSensor Green confirmed that ALO did alter lysosomal acidification in PCa (Figure 2A,B). Temporal analysis showed that the acidified lysosomes then enlarged and fused together, creating larger vacuoles. Bafilomycin A1 (Baf A1), a specific inhibitor of V‐ATPase, can decrease lysosomal acidification. As expected, upon treatment with Baf A1, ALO‐induced lysosomal vacuolation and cell death were markedly inhibited (Figure 2C and Figure S5A–C). Moreover, knockdown of the ATP6V1A (V1A) subunit, a component of the catalytic domain of V‐ATPase, was accompanied by significant inhibition of lysosomal vacuolation and cell death (Figure S5D–G).

The fundamental mechanism regulating V‐ATPase activity involves the reversible disassembly and reassembly of its V1 and Vo domains on the lysosomal membrane [10, 36]. We investigated V‐ATPase assembly by assessing the membrane levels of the V1A subunit relative to those of the V0D subunit. Subcellular fractionation revealed that the membrane‐bound ATP6V1A was significantly increased in ALO‐treated cells (Figure 2D), Co‐immunoprecipitation (Co‐IP) confirmed an increased interaction between ATP6V1A and ATP6V0D1 (Figure 2E). Colocalization analysis showed increased recruitment of ATP6V1A to LAMP1‐positive lysosomes (Figure 2F and Figure S6A) and increased colocalization with ATP6V0D1 (Figure 2G and Figure S6B). Meanwhile, the presence of ATP6V0D1 in the lysosomal was not changed (Figure 2H and Figure S6C). Ratiometric pH measurement indicated that ALO reduced the lysosomal pH in 22Rv1 cells from approximately 4.5–3.5. In normal prostate epithelial RWPE‐1 cells, the pH change was less pronounced (Figure 2I,J and Figure S6D). These data indicate that ALO treatment leads to an enhanced V1‐V0 association, which contributes to lysosomal hyperacidification.

We next evaluated the biochemical consequences of this extreme pH drop. Western blot analysis showed a decrease in mature Cathepsin D (CTSD) levels in ALO‐treated LNCaP and 22Rv1 cells, whereas CTSD processing was maintained in RWPE‐1 cells (Figure 2K). Time‐course analysis in 22Rv1 cells showed that the reduction of mature CTSD coincided with the accumulation of autophagic markers p62 and LC3B‐II (Figure 2L). RWPE‐1 cells did not exhibit significant changes in CTSD or autophagic markers under the same conditions. This suggests that ALO impairs lysosomal degradation in these prostate cancer cells.

To assess the structural integrity of lysosomes, we monitored Galectin‐3 localization. Immunofluorescence imaging showed that ALO treatment resulted in the formation of Galectin‐3 puncta that colocalized with LAMP1 in 22Rv1 cells (Figure 2M). In ALO‐treated RWPE‐1 cells, Galectin‐3 remained diffusely distributed in the cytosol without distinct puncta formation (Figure 2M). Quantitative analysis of Galectin‐3 puncta per cell further supported this observation. These quantitative results support a divergence between RWPE‐1 and 22Rv1 cells. RWPE‐1 cells showed vacuolation without evident Galectin‐3 puncta formation, whereas 22Rv1 cells showed LMP‐associated Galectin‐3 puncta under the same treatment conditions. To define the temporal order of LMP, a time‐course Galectin‐3 puncta assay was performed. 22Rv1 cells were treated with ALO for 0, 4, 8, 12, and 24 h. At 4 h, cells showed evident lysosomal swelling without Galectin‐3 puncta formation (Figure S6E). This observation is consistent with an early adaptive or pre‐LMP response. However, massive Galectin‐3 puncta emerged starting at 8 h. The puncta count peaked at 24 h. This temporal dynamic is consistent with the late‐stage loss of mature Cathepsin D. Together, these time‐resolved data support the interpretation that early vacuolation precedes overt LMP. These findings support a model in which hyperacidification‐associated LMP represents a late execution‐associated event in ALO‐treated prostate cancer cells. These observations support preferential induction of LMP‐associated changes in 22Rv1 cells compared with RWPE‐1 cells under the tested conditions.

### 3.5 Osmotic Stress and Ion Influx Dictate Vacuole Expansion

Although vesicle acidification is essential for membrane fusion, increased osmotic pressure and excessive swelling are also crucial. We hypothesize that although the initial lysosomal acidification facilitates membrane fusion [37], ultimately it will trigger an influx of water, leading to extensive vacuolation [38, 39]. The influx of water partly reduces lysosomal hyperacidification. Consistent with this premise, cells in hypertonic medium containing sorbitol markedly attenuated ALO‐induced lysosomal vacuolation, whereas incubation in hypotonic medium substantially exacerbated it (Figure S7A). Due to a lack of specialized water channels, water movement across the lysosomal membrane is typically driven passively. The transmembrane movement of water is typically driven by osmotic pressure and occurs passively, following the flux of ions, particularly Na^+^ and Cl^−^. Several ion transporters, including Na^+^/H^+^ exchangers (NHEs) and Cl^−^/H^+^ exchangers (CLCs), facilitate the exchange of protons for sodium or chloride ions, leading to the influx of both water and ions into the lysosome [39]. To ascertain whether ion transporters are responsible for the lysosomal vacuolation caused by ALO, EIPA (an NHE inhibitor) or NPPB (a CLC inhibitor) was applied in the co‐culture experiments. Indeed, EIPA or NPPB could block the lysosomal vacuolation induced by ALO treatment (Figure S7A). However, EIPA, NPPB, and hypertonic medium could not inhibit ALO‐induced cell death (Figure S7B–D). This indicates a dissociation between the morphological phenotype and the lethal executioner. Notably, the immunofluorescence results demonstrated that EIPA, NPPB, and hypertonic medium treatment reduced the size of vacuoles but could not completely prevent vacuole formation (Figure S8A,B). The substantial inhibitory effects of EIPA, NPPB, and hypertonic medium on ALO‐induced vacuole formation are likely due to the inhibition of the proton‐driven influx of NaCl and water into lysosomes. Consistently, the knockdown of the ATP6V1A subunit, which inhibits lysosomal V‐ATPase activity, did not prevent the vacuoles induced by hypotonic medium (Figure S8C), suggesting that water influx during lysosomal vacuolation occurs downstream of V‐ATPase activity. Considering these observations, we propose the following mechanistic model: ALO is associated with enhanced V1‐V0 association on lysosomal membranes, which contributes to lysosomal hyperacidification. This extreme acidity not only denatures lysosomal enzymes but also creates a substantial osmotic gradient. The subsequent proton‐driven influx of ions and water leads to significant physical swelling. Ultimately, this combined biochemical and osmotic stress may contribute to LMP and downstream loss of lysosomal integrity.

### 3.6 HSPA8 is Identified as the Critical Functional Node of ALO

**FIGURE 3:**
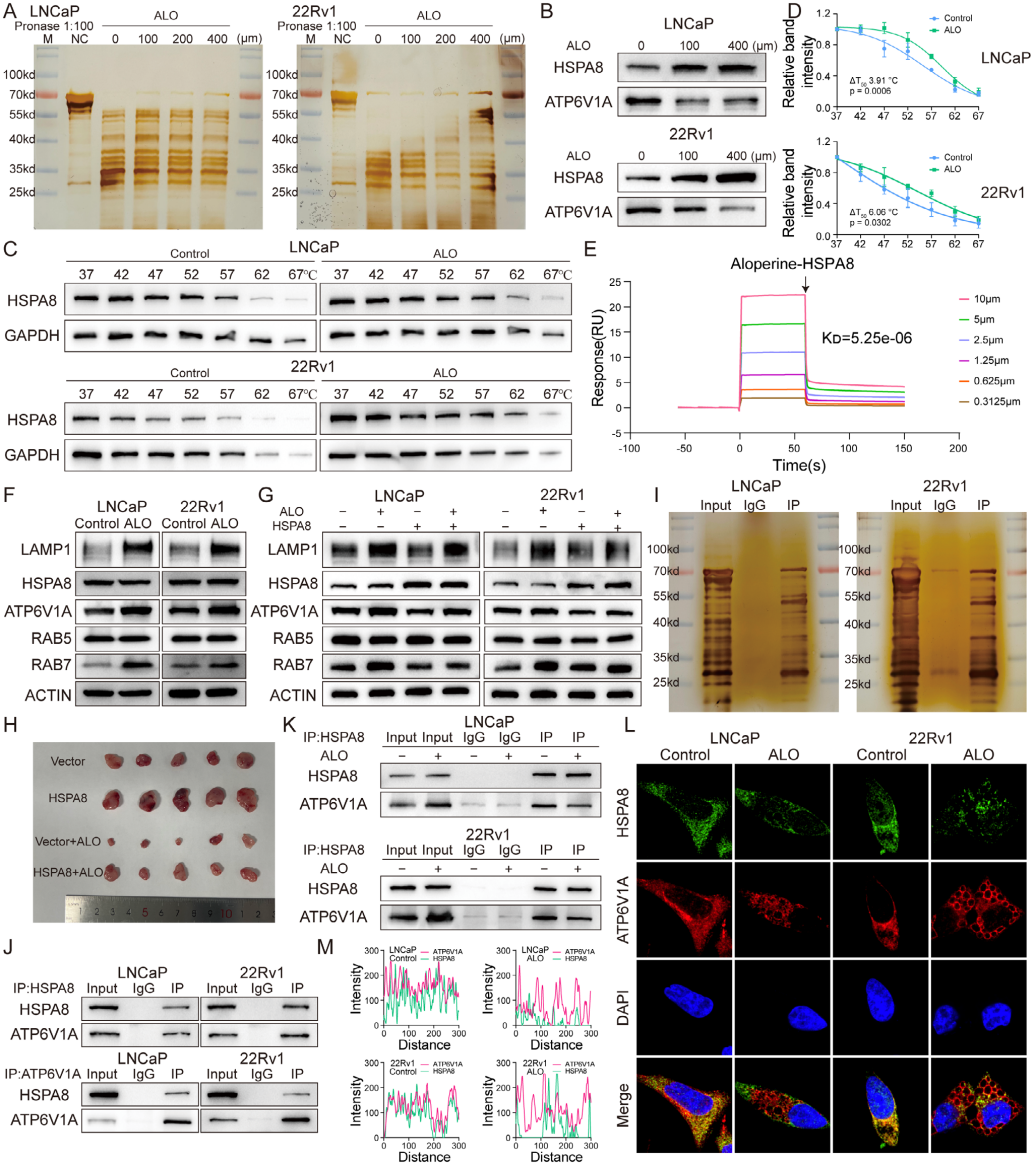
ALO Targets HSPA8 to trigger V‐ATPase hyperassembly. (A) DARTS assays combined with LC–MS/MS analysis identified HSPA8 as a direct target of ALO in LNCaP and 22Rv1 cell lysates. (B) Immunoblotting of DARTS samples showing that ALO protected HSPA8 from proteolysis, confirming their binding. (C, D) CETSAs using LNCaP and 22Rv1 cell extracts provided supportive evidence for the interaction between ALO and HSPA8 (n = 3). (E) SPR sensorgram quantifying the direct binding between ALO and the purified HSPA8 protein. The black arrow indicates the start of the dissociation phase. (F) Immunoblot analysis of the expression of key lysosomal and endosomal proteins in cells treated with 200 µm ALO for 24 h. (G) Effects of ALO on target protein levels upon HSPA8 overexpression. (H) Representative images of tumors excised from xenograft models established with WT or HSPA8‐overexpressing (OE‐HSPA8) 22Rv1 cells and treated with ALO or vehicle (n = 5). (I) Silver staining images of immunoprecipitated complexes. (J) Co‐IP of endogenous HSPA8 and ATP6V1A from cell lysates. (K) Co‐IP analysis showing that ALO treatment disrupts the HSPA8‐ATP6V1A interaction. (L, M) Immunofluorescence analysis and quantification of HSPA8 and ATP6V1A colocalization in cells treated with 200 µm ALO or PBS for 24 h. Data are presented as mean ± SD. ^*^ *p* < 0.05, ^**^ *p* < 0.01, ^***^ *p* < 0.001. Scale bar: 10 µm.

To identify the specific molecular target of ALO that causes lysosomal vacuolation and its anti‐cancer effects in PCa cells, we used the drug affinity‐responsive target stability (DARTS) method. The results showed that a specific protein band, approximately 70 kDa, exhibited dose‐dependent protection against pronase degradation following ALO treatment (Figure 3A). Mass spectrometry (MS) of this band identified peptides corresponding to the V‐ATPase subunit ATP6V1A and the chaperone HSPA8 (Figure S9A–D), which were subsequently verified through immunoblotting (Figure 3B), and the increased immunoblotting signal identified HSPA8 as a critical functional node of ALO. The potential interaction between ALO and HSPA8 was further supported by a Lysate‐based CETSA / thermal shift assay (Figure 3C,D). To further assess the interaction between ALO and HSPA8 and determine its binding affinity, we first performed molecular docking (Figure S9E), which revealed a potential interaction. This interaction was subsequently confirmed by surface plasmon resonance (SPR), which revealed a dissociation constant (KD) of 5.25e‐6 m, confirming direct and moderate binding affinity (Figure 3E).

We hypothesized that HSPA8 is directly affected by ALO‐induced lysosomal vacuolation in 22Rv1 and LNCaP cells. To test this, we examined how ALO treatment affected HSPA8 expression. Initial assessments of HSPA8 expression showed that ALO treatment did not significantly change HSPA8 mRNA or protein levels in prostate tumor cells (Figure 3F and Figure S10A). Western blot analysis showed that ALO significantly increased the expression of the lysosomal markers LAMP1 and RAB7, while the expression of the early endosome marker RAB5 remained unchanged (Figure 3F). To further investigate the molecular mechanism by which ALO targets HSPA8 to promote lysosomal vacuolation, we established cell lines with stable HSPA8 knockdown (designated Vector‐NC, shHSPA8‐1, and shHSPA8‐2) and HSPA8 overexpression (designated Vector‐OE and OE‐HSPA8) for gain‐ and loss‐of‐function assays. The results of the CCK‐8 assay demonstrated that knockdown of HSPA8 expression significantly inhibited the proliferation of 22Rv1 and LNCaP cells, whereas overexpression of HSPA8 resulted in increased proliferation (Figure S10B–H). Western blot analysis revealed that HSPA8 overexpression significantly attenuated the ALO‐induced upregulation of LAMP1 and RAB7 expression (Figure 3G). Additionally, using an animal model with subcutaneous PCa tumors, we confirmed that HSPA8 overexpression promoted prostate tumor growth in vivo (Figure 3H and Figure S10I,J). More importantly, prostate tumor xenografts exhibited reduced sensitivity to ALO when HSPA8 was overexpressed. Crucially, in vitro assays confirmed that HSPA8 overexpression significantly rescued cell viability (Figure S10K,L) and substantially counteracted ALO‐induced lysosomal vacuolation, as evidenced by immunofluorescence (Figure S11A–C). In conclusion, our findings demonstrate that HSPA8 contributes to prostate tumor growth and plays a crucial role in ALO‐induced lysosomal vacuolation in prostate tumors.

### 3.7 ATP6V1A is a CMA Substrate

CMA, a selective autophagy pathway, transports specific cytosolic proteins to lysosomes for degradation [40]. This process depends on the identification of individual substrate proteins. HSPA8 is a crucial initiator and regulator of CMA [40, 41]. By removing damaged proteins, CMA contributes to cellular homeostasis. In cancer cells, CMA inhibition induces proteotoxic stress and metabolic dysregulation. These consequences impede tumor cell proliferation and may result in cell death. We propose that ALO binding to HSPA8 competitively blocks its interaction with CMA substrates containing KFERQ‐like motifs, and this disruption prevents normal clearance of target proteins. To identify potential downstream proteins of HSPA8 involved in this process, we used Co‐IP combined with mass spectrometry to characterize the HSPA8 interactome. This approach successfully identified the ATP6V1A as a putative binding partner of HSPA8 (Figure 3I and Figure S11D,E).

**FIGURE 4:**
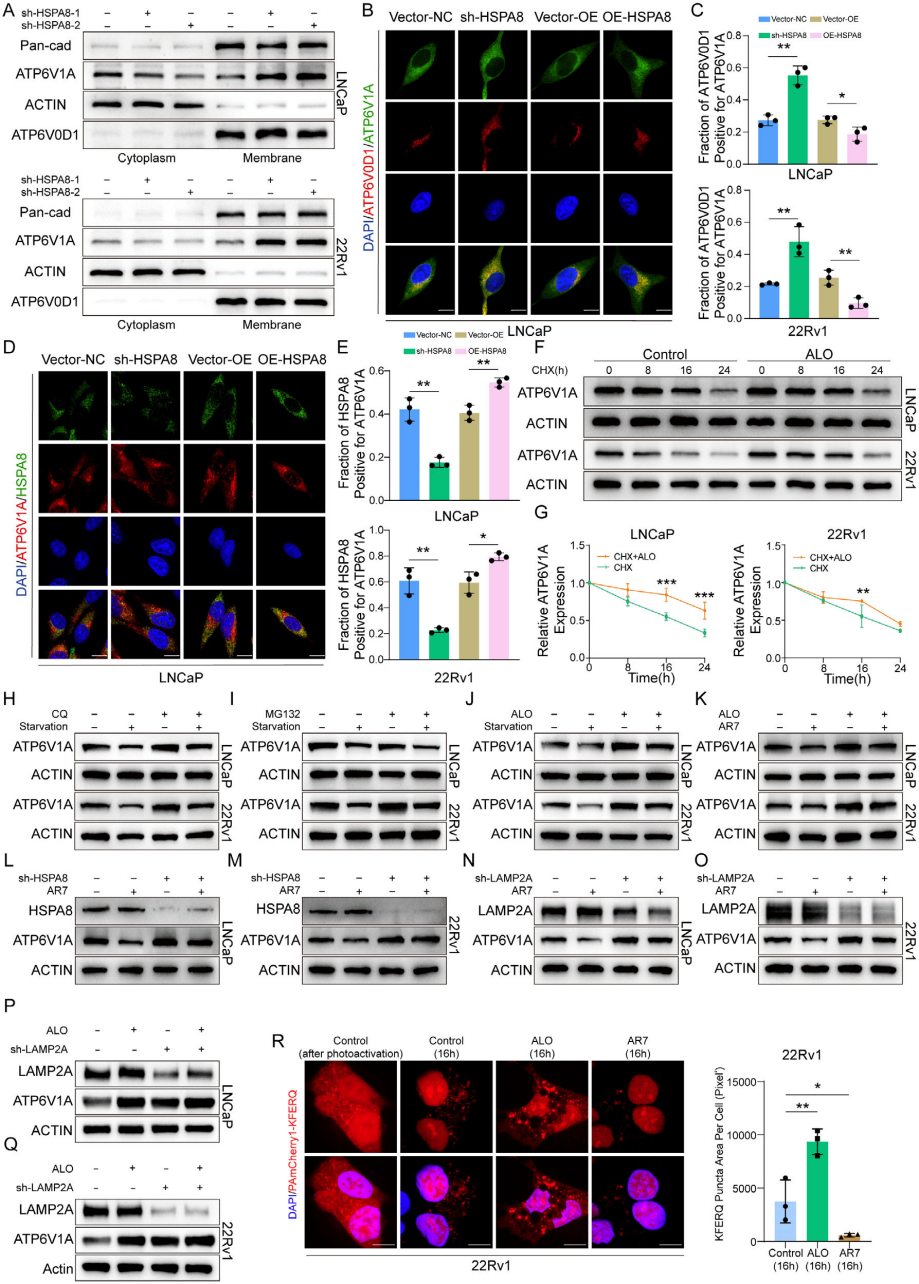
HSPA8 regulates V‐ATPase assembly by mediating the CMA‐dependent degradation of ATP6V1A. (A) Immunoblot analysis of membrane fractions from LNCaP and 22Rv1 cells with HSPA8 knockdown, showing enhanced V1–V0 assembly (increased ATP6V1A/ATP6V0D1 ratio). (B) Immunofluorescence colocalization analysis of ATP6V0D1 and ATP6V1A in LNCaP cells with HSPA8 knockdown or overexpression. (C) Quantification of ATP6V1A recruitment in (B) and Figure S6A (n = 3). (D) Immunofluorescence colocalization analysis of HSPA8 and ATP6V1A in the HSPA8‐knockdown or HSPA8‐overexpressing LNCaP cells. (E) Quantification of ATP6V1A recruitment in (D) and Figure S6B (n = 3). (F, G) Cycloheximide (CHX) chase assays demonstrate that ALO treatment stabilizes the ATP6V1A protein and extends its half‐life in LNCaP and 22Rv1 cells. (H‐K) ATP6V1A protein levels under various conditions: inhibition of lysosomal degradation with CQ (H); inhibition of proteasomal degradation with MG132 (I); under nutrient starvation (J); and with the CMA activator AR7 (K). These results indicate that ATP6V1A is degraded via the CMA pathway and that ALO blocks this process. (L–O) The degradation of ATP6V1A induced by the CMA activator AR7 was abolished upon knockdown of HSPA8 (L, M) or the core CMA receptor LAMP2A (N, O). (P, Q) Immunoblot analysis of ATP6V1A in control and LAMP2A‐knockdown LNCaP (P) and 22Rv1 (Q) cells treated with ALO, assessing epistatic effects. (R) Representative images and quantitative bar charts of 22Rv1 cells expressing the PA‐mCherry1‐KFERQ CMA reporter. Cells were treated with vehicle, ALO, or AR7 for 16 h following photoactivation. The fluorescent puncta area per cell was quantified to evaluate reporter clearance. Data are presented as mean ± SD. ^*^ *p* < 0.05, ^**^ *p* < 0.01, ^***^ *p* < 0.001. Scale bar: 10 µm.

A fundamental criterion for a protein's classification as a CMA substrate is its capacity to directly associate with HSPA8. The physical interaction between HSPA8 and ATP6V1A was validated in LNCaP and 22Rv1 cells via Co‐IP assays (Figure 3J). Moreover, the intracellular colocalization exhibited a robust connection in RWPE‐1, LNCaP, and 22Rv1 cell lines (Figure 3L and Figure S11F). Conversely, ALO treatment significantly diminished the HSPA8‐ATP6V1A interaction, as evidenced by both co‐IP and imaging analyses (Figure 3K,L). Consistent with these findings, knocking down HSPA8 expression (Figure S11G) significantly increased ATP6V1A levels, particularly at the membrane (Figure 4A). To evaluate the clinical correlation of this target, we analyzed prostate cancer patient datasets. HSPA8 expression was significantly elevated in tumors with high Gleason scores compared to normal tissues or lower‐grade tumors (Figure S11H), and high HSPA8 levels correlated with poorer progression‐free survival (PFS) (Figure S11I). Notably, a strong positive correlation was observed between HSPA8 and ATP6V1A expression in clinical cohorts (Figure S11J), underscoring their pathological relevance. Furthermore, we demonstrated that the absence of HSPA8 augmented the colocalization of ATP6V1A and ATP6V0D1, concurrently diminishing their association with HSPA8. Conversely, this trend was reversed upon the overexpression of HSPA8 (Figure 4B–E and Figure S12A,B).

Since the modulation of HSPA8 expression did not alter the transcription of ATP6V1A (Figure S12C,D), we hypothesized that the change in ATP6V1A levels might be due to altered protein degradation dynamics. To confirm this, we used cycloheximide (CHX) to block protein synthesis in the cells and monitored the remaining ATP6V1A at different time points after ALO treatment (Figure 4F,G). The results demonstrated a significantly prolonged half‐life of ATP6V1A in ALO‐treated cells. Based on previous studies establishing the essential role of ATP6V1A in V‐ATPase hyperassembly‐induced lysosomal vacuolation, we propose a molecular mechanism for ALO activity. Our results suggest that ALO binding to HSPA8 competitively disrupts the normal HSPA8‐ATP6V1A interaction. This interference subsequently impairs ATP6V1A clearance via lysosomal degradation pathways.

Since numerous studies have reported CMA activation under conditions such as starvation and oxidative stress [42, 43], we subjected PCa cells to serum starvation and observed a time‐dependent decrease in ATP6V1A protein levels (Figure S12E,F). To identify the pathway responsible for ATP6V1A degradation, we inhibited the proteasome with MG132 and lysosomes with CQ (Figure 4H,I). MG132 treatment had no effect on ATP6V1A levels. In contrast, CQ‐mediated inhibition of lysosomal hydrolases significantly increased ATP6V1A expression and partially abolished the starvation‐induced reduction in ATP6V1A levels. This stabilization occurred without altering ATP6V1A transcription (Figure S12I,J).

Consistently, ALO treatment also reversed the starvation‐induced decrease in ATP6V1A expression (Figure 4J). We then tested whether ALO could counteract the effects of CMA activation. When we cotreated PCa cells with the CMA‐specific agonist AR7 [44] and ALO, we found that ALO effectively blocked the AR7‐mediated reduction in ATP6V1A protein levels (Figure 4K). In addition, knocking down lysosomal‐associated membrane protein 2A (LAMP2A) [45, 46], another core CMA component, also stabilized ATP6V1A by attenuating its degradation (Figure S12K,L). Importantly, the downregulation of ATP6V1A induced by AR7 was reversed by knockdown of either HSPA8 or LAMP2A (Figure 4L–O). To further confirm that ALO targets this specific degradative axis, we assessed ATP6V1A levels following ALO treatment in combination with LAMP2A knockdown. While both ALO treatment and LAMP2A depletion independently induced ATP6V1A accumulation, their combination did not exhibit a synergistic effect (Figure 4P,Q), indicating that they operate within the same CMA pathway. Finally, to functionally monitor global CMA activity, we utilized the photoactivatable CMA reporter PA‐mCherry1‐KFERQ. Following photoactivation, the degradation of the fluorescent reporter was visibly delayed in ALO‐treated cells compared to control cells, whereas the CMA agonist AR7 accelerated its clearance. Quantitative analysis revealed a significant increase in the residual puncta area in ALO‐treated cells compared to the control. Conversely, the CMA agonist AR7 accelerated the clearance (Figure 4R and Figure S12M,N). Together, these results demonstrate that ALO increases ATP6V1A levels by inhibiting HSPA8‐dependent CMA activity.

**FIGURE 5:**
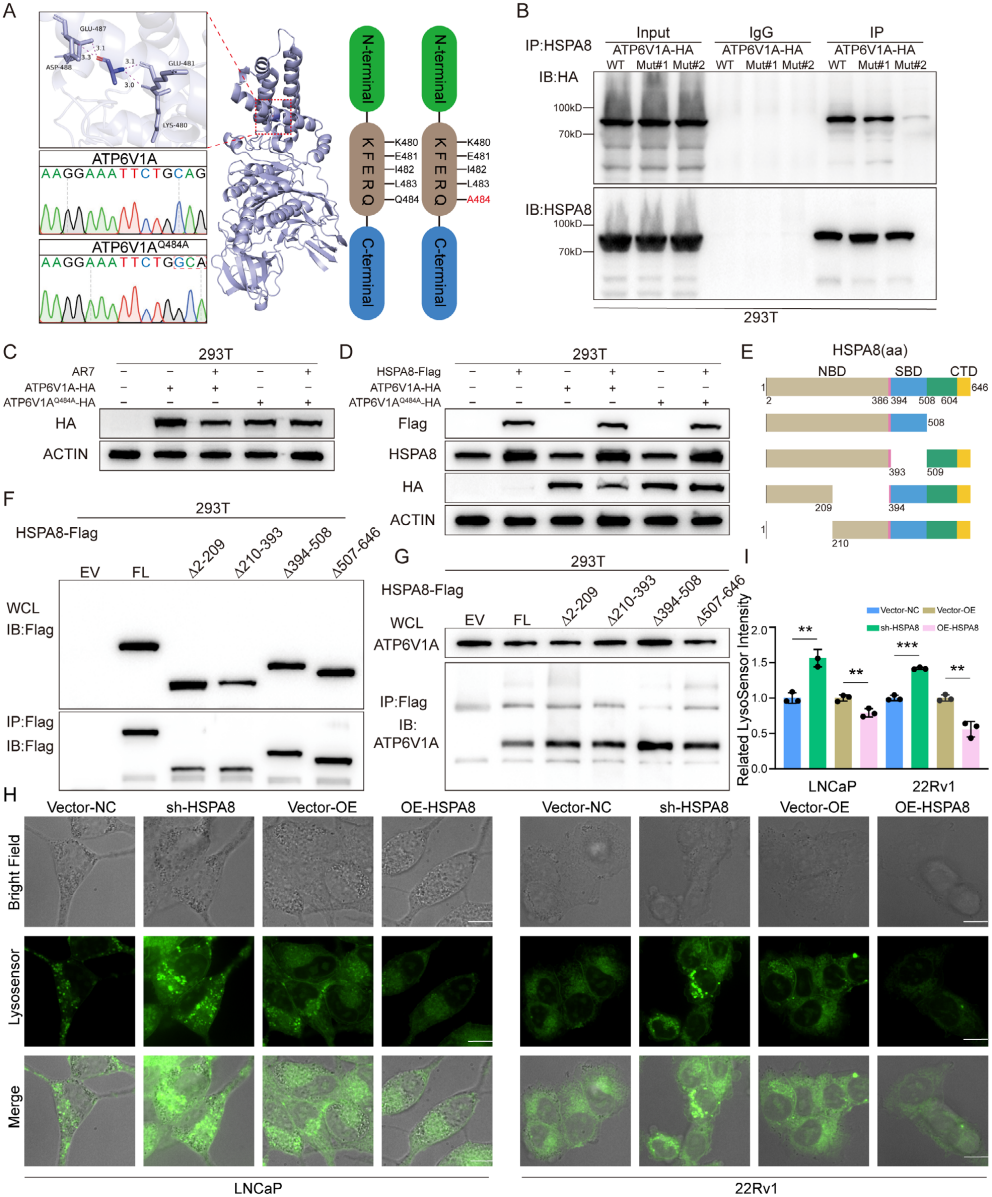
The pentapeptide sequence ^480^KEILQ^484^ is critical for CMA‐mediated degradation. (A) Surface representation of ATP6V1A with the KFERQ motif (^480^KEILQ^484^), highlighted in red, exposed for HSPA8 accessibility. (B) HEK 293 cells were transfected with ATP6V1A^Q484A^‐HA for 48 h. Lysates were immunoprecipitated (IP) with either IgG or an anti‐HA antibody and probed for ATP6V1A^Q484A^‐HA and HSPA8. (C) HA expression in HEK 293 cells transfected with ATP6V1A^Q484A^‐HA in the presence of AR7 for 48 h. (D) HA expression in HEK 293 cells with HSPA8‐Flag overexpression, transfected with ATP6V1A^Q484A^‐HA for 48 h. (E) Schematic representation of FLAG‐tagged HSPA8 constructs (full‐length and truncation mutants) used to map the ATP6V1A‐binding domain. (F, G) Co‐IP assays in HEK293T cells transfected with the indicated HSPA8‐FLAG constructs. Cell lysates were immunoprecipitated with anti‐FLAG beads, and bound ATP6V1A was detected by immunoblotting, which revealed the domain essential for the interaction between HSPA8 and ATP6V1A. (H) Representative fluorescence images of LNCaP and 22Rv1 cells with HSPA8 knockdown or overexpression stained with LysoSensor Green to assess lysosomal acidity. (I) Quantification of LysoSensor Green fluorescence intensity from (H) (n = 3). Data are presented as mean ± SD. ^*^ *p* < 0.05, ^**^ *p* < 0.01, ^***^ *p* < 0.001. Scale bar: 10 µm.

These findings, taken together, indicate that ALO elevates ATP6V1A levels by suppressing HSPA8‐mediated CMA activity. Proteins targeted for degradation via CMA generally possess a canonical KFERQ motif, which functions as the recognition site for HSPA8. Employing the KFERQ Finder V0.8 web server [47], we discovered two KFERQ‐like motifs within ATP6V1A: ^231^QRVLD^235^ and ^480^KEILQ^484^. Given that the Q‐to‐A mutation effectively abrogated CMA substrate recognition by HSPA8, we constructed two HA‐tagged mutants, ATP6V1A^Q231A^‐HA and ATP6V1A^Q484A^‐HA, to evaluate the functional significance of these motifs (Figure 5A and Figure S12O). Co‐IP assays demonstrated that compared with wild‐type ATP6V1A, the ATP6V1A^Q484A^ mutant exhibited significantly reduced binding to HSPA8 (Figure 5B). We next investigated the Q484A mutant's response to CMA activation. The CMA agonist AR7 reduced the protein levels of wild‐type ATP6V1A, but ATP6V1A^Q484A^ protein levels did not change (Figure 5C). Similarly, overexpressing HSPA8 increased the degradation of wild‐type ATP6V1A, while the Q484A mutant showed no such effect (Figure 5D). These results together suggest that the ^480^KEILQ^484^ motif is crucial for the CMA‐mediated degradation of ATP6V1A, confirming ATP6V1A as a bona fide CMA substrate. Following this, we studied the interaction between HSPA8 and ATP6V1A, using truncated, epitope‐tagged HSPA8 constructs (Figure 5E). Co‐IP assays revealed that the substrate‐binding domain (SBD) of HSPA8 is necessary for its interaction with ATP6V1A (Figure 5F,G). These findings confirm the functional relationship between HSPA8 and ATP6V1A. To determine how the interaction between HSPA8 and ATP6V1A influences lysosomal vacuolation, we measured changes in lysosomal pH using LysoSensor. Reducing the HSPA8 level significantly increased the intracellular green fluorescence intensity and lysosomal volume, whereas increasing the HSPA8 level had the opposite effects (Figure 5H,I). Our findings establish a hyperacidification‐driven osmotic model where the uncontrolled assembly of V‐ATPase holoenzymes transforms the lysosome from a degradative hub into a severe osmotic sink.

### 3.8 Lysosomal Stress Alters the Cholesterol Metabolic Profile and its Spatial Distribution

**FIGURE 6:**
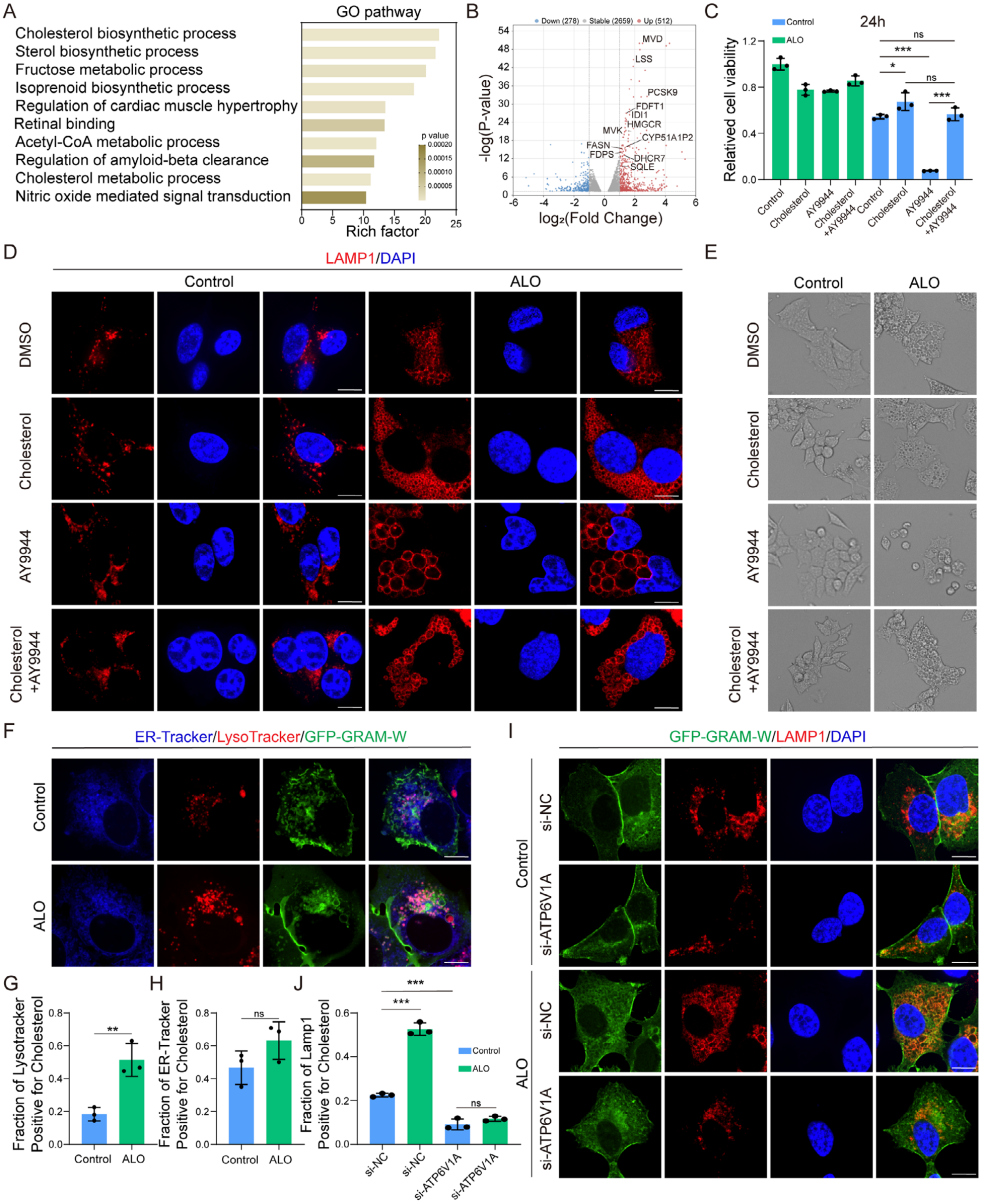
V‐ATPase hyperactivation promotes cholesterol transport. (A) Gene Ontology (GO) Enrichment Analysis of differentially expressed genes in 22Rv1 cells treated with 200 µm ALO or PBS for 24 h, presented as a bubble chart. (B) Volcano plot illustrating genes involved in cholesterol biosynthesis pathways that are differentially expressed following ALO treatment. (C) LNCaP and 22Rv1 cell viability was assessed by a CCK‐8 assay after treatment with 200 µm ALO, 50 µg/mL water‐soluble cholesterol, 1 µm AY9944 (a cholesterol synthesis inhibitor), or their combination for 24 h (n = 3). (D) Immunofluorescence staining of LAMP1 in 22Rv1 cells under the same treatment conditions as in (C). (E) Bright field microscopy images showing morphological changes in 22Rv1 cells under the indicated treatments. (F) Cholesterol is recruited to lysosomes upon treatment with 200 µm ALO, as visualized by immunofluorescence microscopy. (G, H) Quantification of cholesterol recruitment to lysosomes (G) and the ER (H) after treatment as indicated in (F) (n = 3). (I) Immunofluorescence analysis of GFP‐GRAM‐W (a cholesterol sensor) and LAMP1 expression in ATP6V1A‐knockdown 22Rv1 cells with or without ALO treatment. (J) Quantification of cholesterol recruitment to lysosomes in (I) (n = 3). Data are presented as mean ± SD. ^*^ *p* < 0.05, ^**^ *p* < 0.01, ^***^ *p* < 0.001. Scale bar: 10 µm.

Lysosomal vacuolation involves a physical expansion of the limiting membrane. To elucidate how prostate cancer cells transcriptionally adapt to this sudden increase in membrane surface area driven by V‐ATPase hyperassembly, we performed RNA sequencing on 22Rv1 cells treated with ALO for 24 h. This analysis showed consistent and significant changes in gene expression. The most activated pathways were related to cholesterol biosynthesis, sterol synthesis, and cholesterol metabolism (Figure 6A). The expression levels of crucial genes implicated in de novo lipogenesis and cholesterol uptake [48], such as FASN, FDPS, SQLE, HMGCR, MVD, MVK, IDI1, FDFT1, LSS, CYP51A1, DHCR7, and PCSK9, exhibited significant increases (Figure 6B and Figure S13A). Previous research has demonstrated that the subcellular localization of V‐ATPase influences cholesterol trafficking; specifically, elevated V‐ATPase expression on endosomes facilitates cholesterol transport via enhanced endosomal acidification [36, 49, 50]. In accordance with this premise, we propose that the augmented V‐ATPase assembly observed during ALO‐induced lysosomal vacuolation may support the expression and functionality of de novo fatty acid synthesis pathways within prostate tumor cells, consequently affecting cholesterol metabolic homeostasis. To evaluate this hypothesis, we modulated cholesterol levels in prostate tumor cells and subsequently examined their responses to ALO treatment (Figure 6C and Figure S13B,C). The DHCR7 inhibitor AY9944 [51] increased the sensitivity of cells to ALO, whereas the addition of water‐soluble cholesterol increased their resistance. Although neither treatment prevented lysosomal vacuolation (Figure 6D,E), different effects on vacuole morphology were observed: AY9944 created fewer but larger LAMP1‐positive vacuoles, whereas water‐soluble cholesterol generated more numerous but smaller vacuoles. This difference may reflect how cells combat osmotic stress; when more cholesterol is available, the cells may shuttle it to swollen lysosomes to help manage changes in pressure.

To further investigate how cholesterol moves, we used a GFP‐GRAM‐W cholesterol probe to observe its movement in real time. The results showed that ALO treatment significantly changed where cholesterol was located on lysosomal membranes. Specifically, we saw a strong overlap between LAMP1‐labeled vacuoles and GFP‐GRAM‐W fluorescence signals, and the cholesterol/lysosome ratio increased significantly (Figure 6F–H). These findings suggest that V‐ATPase hyperactivation may alter the lipid environment of the lysosomal membrane, leading to a specific increase in cholesterol in these areas. In contrast, the cholesterol/ER ratio remained constant, indicating that ALO promotes cholesterol synthesis and contributes to cholesterol accumulation in lysosomes. Following ATP6V1A knockdown, the subsequent inhibition of V‐ATPase function reversed both lysosomal cholesterol accumulation and vacuolation (Figure 6I,J). These results strongly support the hypothesis that V‐ATPase acts as a central regulator of the spatial distribution of cholesterol. Consequently, we suggest that ALO treatment leads to an increase in V‐ATPase assembly‐related readouts, inducing lysosomal hyperacidification, which alters membrane lipid fluidity and enhances directional cholesterol transport to lysosomal membranes. The consequent abnormal accumulation of cholesterol within lysosomes further exacerbates vacuolation, thereby establishing a positive feedback loop. In summary, these observations elucidate a pathological mechanism: ALO‐driven V‐ATPase hyperactivity not only directly instigates lysosomal vacuolation but also remodels the spatial distribution of cholesterol, initiating a metabolic adaptation that ultimately renders tumor cells reliant on the cholesterol biosynthetic pathway.

### 3.9 PDZD8 Mediates ALO‐Induced Lysosomal Vacuolation

**FIGURE 7:**
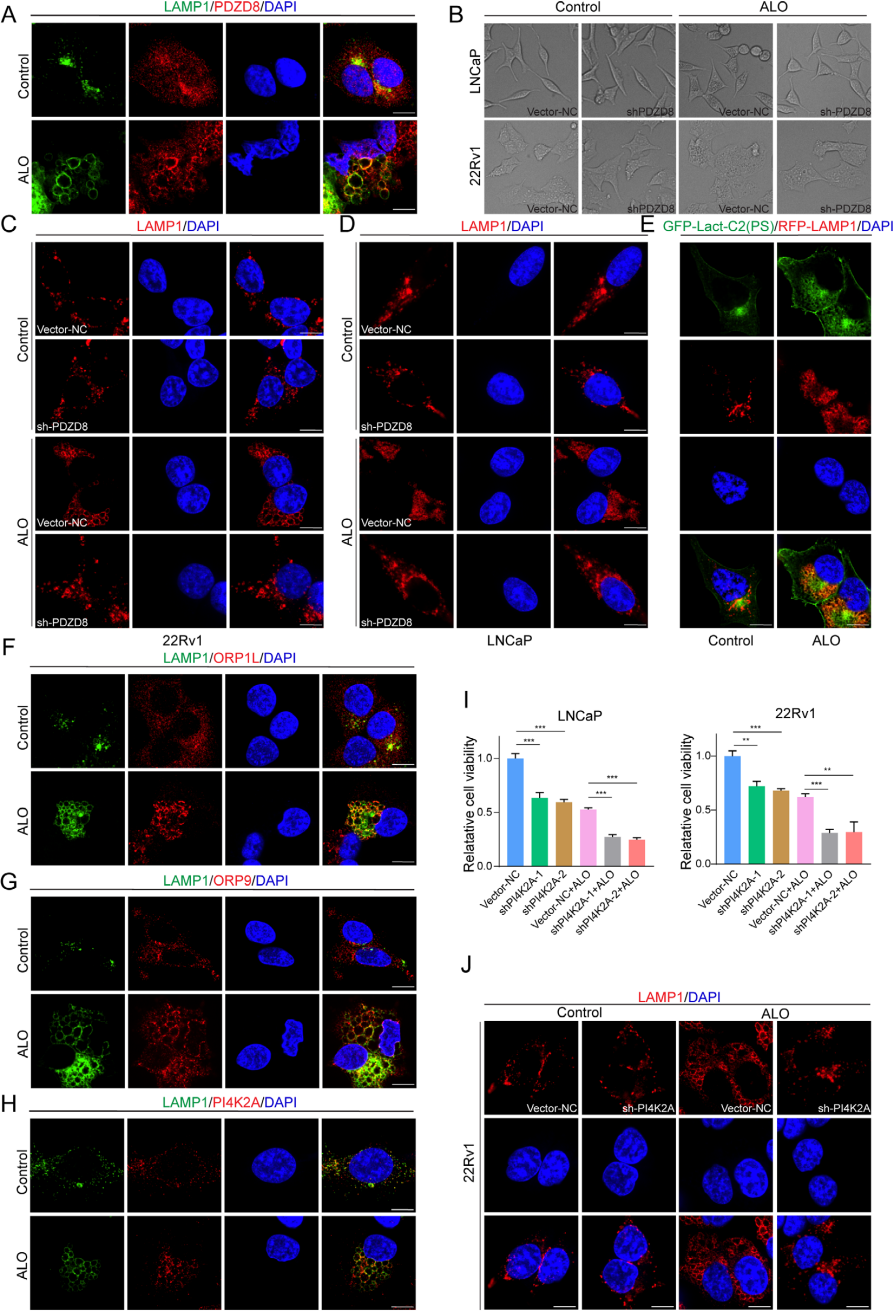
PDZD8 mediates ALO‐induced lysosomal vacuolation. (A) PDZD8 is recruited to lysosomes upon treatment with 200 µm ALO, as shown by immunofluorescence microscopy. (B) Microscopy images of ATP6V1A‐knockdown LNCaP and 22Rv1 cells treated with 200 µm ALO. (C, D) Immunofluorescence staining of LAMP1 in ATP6V1A‐knockdown LNCaP (D) and 22Rv1 (C) cells treated with 200 µm ALO. (E) PS is recruited to lysosomes upon treatment with 200 µm ALO, as shown by immunofluorescence microscopy. (F) ORP1L is recruited to lysosomes upon treatment with 200 µm ALO, as shown by immunofluorescence microscopy. (G) ORP9 is recruited to lysosomes upon treatment with 200 µm ALO, as shown by immunofluorescence microscopy. (H) PI4K2A is recruited to lysosomes upon treatment with 200 µm ALO, as shown by immunofluorescence microscopy. (I) The viability of PI4K2A‐knockdown LNCaP and 22Rv1 cells treated with 200 µm ALO for 24 h was determined by a CCK‐8 assay (n = 3). (J) Immunofluorescence staining of LAMP1 in PI4K2A‐knockdown 22Rv1 cells treated with 200 µm ALO for 24 h. Data are presented as mean ± SD. ^*^ *p* < 0.05, ^**^ *p* < 0.01, ^***^ *p* < 0.001. Scale bar: 10 µm.

Recent studies have identified PDZD8 as a common mediator of lysosomal vacuolation induced by various pharmacological agents [52]. To determine whether PDZD8 is essential for ALO‐induced lysosomal vacuolation, we generated PDZD8‐knockdown cells (shPDZD8‐1 and shPDZD8‐2) (Figure S13D–F). Notably, PDZD8 deficiency completely abolished ALO‐induced lysosomal vacuolation (Figure 7A–D). Given that PDZD8 is selectively recruited to phosphatidylserine (PS)‐ and cholesterol‐enriched membranes, where it activates membrane tethering and lipid transfer [51, 53, 54, 55] considering our previous findings of cholesterol enrichment in lysosomal vacuolar regions, we further examined PS localization using a GFP‐Lact probe. Our results demonstrated that PS was also recruited to lysosomal vacuoles following ALO treatment (Figure 7E).

Multiple lipid transfer proteins belonging to the oxysterol‐binding protein (OSBP)‐related protein (ORP) family serve as key factors that exchange phosphatidylinositol 4‐phosphate (PI (4) P) for PS and cholesterol (e.g., ORP1L is a PI (4) P/cholesterol transporter and ORP9 is a PI (4) P/PS transporter) [53, 54, 55, 56]. IF analysis revealed the accumulation of several ORPs on vacuolar membranes after their formation (Figure 7F,G). Since ORPs are phosphoinositide effectors typically recruited to PI (4) P‐enriched membranes, we investigated phosphatidylinositol 4‐kinase 2α (PI4K2A) [54, 57], the enzyme responsible for PI (4) P production. Notably, PI4K2A was enriched on lysosomes following ALO treatment (Figure 7H), and its knockdown significantly suppressed ALO‐induced lysosomal vacuolation (Figure 7J and Figure S13G–K), which is consistent with its position upstream of ORPs. Consistent with our previous findings in which various lysosomal ion channel inhibitors were used, knockdown of either PDZD8 or PI4K2A also failed to rescue ALO‐induced cell death (Figure S13L,M). These findings further support the concept that lipid transfer in response to lysosomal osmotic stress acts as a downstream executioner of lysosomal hyperacidification. Our experimental evidence confirms that ALO‐induced lysosomal osmotic stress drives vacuolation through a PDZD8‐dependent mechanism.

## 4 Discussion

ADT significantly improves the survival rate of PCa patients [3]. Conversely, most patients eventually develop resistance to these treatments [58]. Therefore, there is a critical need to develop therapeutic strategies for prostate cancer that are based on non‐apoptotic cell death. Cytoplasmic vacuolation, a distinctive morphological hallmark of non‐apoptotic cell death, is considered a potential therapeutic direction to overcome drug resistance [13, 59]. These vacuoles can come from different organelles, such as mitochondria, the endoplasmic reticulum, endosomes, or lysosomes. Notably, lysosomes and their related autophagy pathways have been shown to significantly contribute to the development of resistance in prostate cancer [5]. In contrast, the clinical usefulness of traditional lysosomal inhibitors, such as CQ, has been restricted [60, 61]. Therefore, specifically inducing lysosomal vacuolation could be a potential therapeutic strategy for patients with PCa.

Previous studies have identified ALO as a potential autophagy modulator [22, 24, 25]. However, its specific role in PCa is still unclear. Our research shows that simply describing ALO as an autophagy inhibitor is insufficient to explain its anticancer mechanisms. We observed that ALO causes significant vacuolation in the cytoplasm. The observed effect persisted despite the application of various substances, encompassing both autophagy inhibitors and methuosis inhibitors. Confocal microscopy and TEM revealed that these vacuoles originated from lysosomes, rather than from autophagy, as illustrated in Figure 1.

Vacuolation‐induced lysosomal dysfunction, observed under diverse conditions, is a fundamental factor in lysosomal storage diseases, Alzheimer's disease, and Parkinson's disease [17, 62, 63]. We have identified ALO as a previously unrecognized inducer of lysosomal vacuolation. Addressing the paradoxical relationship between hyperacidification and enzymatic inhibition is crucial for understanding ALO's mechanism. While lysosomal hydrolases (e.g., Cathepsins) physiologically require an acidic environment for optimal processing and activity (typically pH 4.5–5.0), the hyperacidification (pH ∼3.5) driven by ALO‐induced V‐ATPase hyperassembly likely surpasses this optimal functional range, leading to the denaturation or misfolding of these essential enzymes.

Lysosomal vacuolation results from osmotic stress caused by increased luminal solute load, water influx, and membrane swelling [32, 39, 64]. While previous studies have primarily associated lysosomal osmotic stress with weak lysosomotropism bases or PIKfyve kinase inhibition [19, 32, 52], our results show an assembly‐associated increase in V‐ATPase following ALO treatment, characterized by the enhanced association of the V1 and V0 domains, thereby leading to the formation of more functional holoenzyme complexes. Concurrently, the initial acidification establishes a strong proton gradient that triggers Na^+^/H^+^ and Cl^−^/H^+^ exchangers, causing a substantial influx of NaCl and water into the lysosomal lumen. This water influx results in significant lysosomal swelling, which consequently causes a significant physical dilution of any remaining active enzymes. Biochemical denaturation combined with physical dilution elucidates why ALO induces a complete blockade of substrate degradation and autophagic flux despite establishing a highly acidic environment.

The V‐ATPase complex, which is essential for intracellular pH regulation, functions through a cycle of disassembly and reassembly [10, 16, 65, 66], the precise mechanisms governing this process remain incompletely elucidated. Our study provides new perspectives that HSPA8 is indispensable for preserving normal V‐ATPase activity by modulating ATP6V1A degradation through CMA. The interaction between ALO and HSPA8 impedes the latter's association with ATP6V1A. Consequently, the usual degradation of ATP6V1A is inhibited, resulting in an accumulation of V‐ATPase on lysosomal membranes. Although previous studies have reported that inhibiting HSPA8 produces antitumor effects, we are the first to demonstrate that disruption of HSPA8 induces lysosomal vacuolation in PCa. HSPA8 knockdown suppressed PCa cell proliferation, whereas its overexpression attenuated ALO‐induced lysosomal vacuolation, indicating that the anticancer activity of ALO is dependent on HSPA8. CMA is a selective process where HSPA8 identifies misfolded proteins for internalization via LAMP2A [44, 45, 67]. Our initial evidence suggesting CMA's role in ATP6V1A regulation came from the finding that HSPA8 directly interacts with and promotes the breakdown of ATP6V1A. LAMP2A, an isoform created by alternative splicing of the LAMP2 gene, is the only one linked to CMA. When LAMP2A was selectively silenced, ATP6V1A levels increased, highlighting CMA's specific role in regulating ATP6V1A. The KFERQ pentapeptide motif in substrate proteins is essential for CMA‐mediated degradation, acting as the recognition site for HSPA8 binding [68]. We found the ^480^KEILQ^484^ pentapeptide in ATP6V1A and showed that modifying to this sequence significantly impaired the ATP6V1A–HSPA8 interaction, confirming that ATP6V1A is a bona fide CMA substrate.

Furthermore, our data clarify the role of cholesterol metabolism in lysosomal vacuolation, which serves as a compensatory cellular adaptive response. During ALO treatment, vacuolar membranes grow very large, which means they need a constant supply of structural lipids. Consequently, ALO‐induced vacuolation causes an increase in cholesterol biosynthesis to protect the quickly growing membranes from osmotic stress. Adding exogenous cholesterol enhances lysosomal membrane tolerance, which makes the cell more resistant. On the other hand, inhibiting cholesterol synthesis with the DHCR7 inhibitor AY9944 takes away the essential lipid pool from the cells, which speeds up membrane failure when osmotic tension is applied. This synergistic lethality underscores a targetable metabolic susceptibility: preemptively disrupting this lipid‐based adaptive mechanism enhances the sensitivity of PCa cells to ALO‐induced osmotic stress.

Recent reports suggest that PDZD8 is a crucial mediator of lysosomal vacuolation triggered by various stimuli, such as PIKfyve inhibition, weak lysosomotropic bases, storage substrates, and hypotonic stress [52]. Consistent with this, we confirmed that PDZD8 is essential for the physical fusion and structural expansion of lysosomes into macro‐vacuoles during ALO‐induced hyperacidification. Intriguingly, while the depletion of PDZD8 effectively abolished the morphological manifestation of lysosomal vacuolation, it failed to rescue ALO‐induced cytotoxicity. This observation indicates that vacuolation acts as a morphological trajectory of osmotic stress, while the true “lysosomal catastrophe” is caused by biochemical instability. By suppressing the structural expansion, PDZD8 knockdown only removes the “visual phenotype” without resolving the underlying biochemical catastrophe that caused it. As evidenced by our Galectin‐3 puncta analysis, the commitment to cell death is primarily driven by the irreversible LMP and subsequent enzymatic dysfunction triggered by lethal hyperacidification, rather than the physical expansion of the vacuoles themselves.

Building upon this LMP‐driven mechanism, it is crucial to position ALO within the broader context of current translational efforts. The translation of lysosome‐ and autophagy‐targeting strategies into the clinic has been an area of intense investigation, most notably with the use of hydroxychloroquine (HCQ) and its derivatives in solid tumors, including prostate cancer. However, the clinical efficacy of HCQ is frequently limited by dose‐limiting toxicities and a lack of tumor specificity. Mechanistically, HCQ functions primarily as a late‐stage autophagy inhibitor by alkalizing the lysosomal lumen and blocking autophagosome‐lysosome fusion. In contrast, ALO presents a distinct mechanistic depth. By disrupting the ATP6V1A‐HSPA8 interaction, ALO induces an opposing physiological effect: the hyper‐acidification of the lysosome, which culminates in extensive vacuolation and LMP.

ALO is mechanistically distinct and shows preliminary selectivity. Prostate cancer cells typically possess a lower basal lysosomal pH and rely heavily on elevated levels of V‐ATPase (ATP6V1A) and chaperones (HSPA8) to maintain lysosomal integrity under metabolic stress. By exploiting this pre‐existing vulnerability, ALO may preferentially push 22Rv1 cells toward LMP‐associated lysosomal damage under the tested conditions. In comparison, RWPE‐1 cells showed less pronounced lysosomal hyperacidification and lacked evident Galectin‐3 puncta formation. These findings suggest preliminary in vitro selectivity of ALO relative to HCQ under the tested conditions. Despite these advantages, the clinical development of ALO will face translational challenges. Establishing the safety and systemic tolerability of LMP‐inducing agents in vivo is paramount, as off‐target lysosomal rupture could lead to normal tissue toxicity. Furthermore, maximizing the clinical efficacy of this approach will require rigorous patient stratification. Given our findings, prostate cancer patients whose tumors exhibit high baseline expression of ATP6V1A and HSPA8, or those characterized by high metabolic acidosis, may be the most susceptible to ALO treatment. Future clinical efforts should prioritize identifying these specific biomarker signatures to effectively translate this LMP‐driven strategy into targeted therapies.

## 5 Conclusions

In conclusion, our study identifies the natural alkaloid ALO as a potent anticancer agent that induces a mode of cell death in PCa via lysosomal hyperacidification. By competitively binding to HSPA8, ALO disrupts the CMA‐dependent turnover of ATP6V1A, leading to enhanced V‐ATPase V1‐V0 association. This molecular event may transform the lysosome into an osmotic sink and contribute to LMP‐associated loss of lysosomal integrity. Furthermore, elucidating compensatory cholesterol biosynthesis triggered by lysosomal hyperacidification and vacuolation uncovers a targetable metabolic susceptibility. These findings not only reshape our comprehension of lysosomal stress trajectories but also underscore the HSPA8‐ATP6V1A axis as a highly potential therapeutic target for addressing drug resistance in advanced prostate cancer.

## Author Contributions

Bingzheng An, **Zhiqing Fang**, and Ze Gao conceived and designed the research. Bingzheng An performed most of the biochemical and molecular experiments, with assistance from Ze Gao, **Shuo Chen**, **Chen Zhang**, **Haochen Cui**, and **Kefan Song**. Animal experiments were performed by Bingzheng An, Ze Gao, and **Liwei Meng**. Bingzheng An, **Lei Yan**, and Zhiqing Fang contributed to the data discussion. Bingzheng An, Ze Gao, and Zhiqing Fang wrote the manuscript. All the authors read and approved the manuscript.

## Funding

This work was supported by grants from the Shandong Provincial Natural Science Foundation (No. ZR2023QH192 and No. ZR2022QH354) and the Shandong University Clinical Research (No. 26010112002340).

## Conflicts of Interest

The authors declare no conflicts of interest.

## Supporting information




**Supporting File 1**: advs76165‐sup‐0001‐SuppMat.docx.

**Supporting File 2**: advs76165‐sup‐0002‐SuppMat.docx.

## Data Availability

The data that support the findings of this study are available from the corresponding author upon reasonable request.
