# Supplementary material for: Targeting the HSPA8‐CMA‐ATP6V1A Axis Triggers Lysosomal Hyperacidification and Catastrophic Vacuolation in Prostate Cancer
Source: Adv Sci (Weinh). 2026 Jun 19;13(51):e76165. doi: 10.1002/advs.76165 (PMC13336817; doi:10.1002/advs.76165)
Supplement: Supplementary file 1 [file ADVS-13-e76165-s001.docx]

 **Supplementary Figure S1. ALO inhibits cancer cell viability and induces morphological vacuolation with lower toxicity in normal cells.** **(A)** Relative cell viability of LNCaP and 22Rv1 cells treated with the indicated concentrations of ALO for 48 h and 72 h, measured by CCK-8 assay. **(B)** Cell viability of normal prostate epithelial RWPE-1 cells treated with ALO for 48 h. The IC50 value is indicated. **(C, D)** Cell viability of 22Rv1 (C) and RWPE-1 (D) cells treated with varying concentrations of chloroquine (CQ) for 48 h. **(E)** Colony formation assay of RWPE-1 cells treated with ALO at the indicated concentrations. **(F)** Bright-field images showing the time-course morphological changes in RWPE-1 and 22Rv1 cells treated with ALO (0 to 48 h). The rightmost panels show cells after a 24 h ALO treatment followed by a 24 h wash-out in drug-free medium. **(G)** Bright-field images of multiple cell lines (786-O, A549, B16-F1, HCT116, LN229, MCF12A, T24) treated with ALO or vehicle control. Data are presented as mean ± SD. *p < 0.05, **p < 0.01, ***p < 0.001. Scale bar: 10 μm. **Supplementary Figure S2. Effects of ALO treatment on hematological and serum biochemical profiles in mice.** **(A)** Quantitative analysis of routine hematological parameters in peripheral blood, including white blood cells (WBC), red blood cells (RBC), hemoglobin (HGB), platelets (PLT), and their related differentials and indices. **(B)** Evaluation of serum biochemical markers assessing hepatic function, renal function, lipid and glucose metabolism, and myocardial enzymes. n=3, Data are presented as mean ± SD. **Supplementary Figure S3. *In vivo* safety evaluation and cellular effects of ALO. (A)** Representative hematoxylin and eosin (H&E) staining images of major organs (lung, liver, spleen, kidney, and heart) harvested from mice in the Control, ALO-Low, and ALO-High groups. **(B)** Body weight changes of mice in the indicated treatment groups on day 7 and day 17. Data are presented as mean ± SD (n = 6 per group). **(C)** Uptake of the fluid-phase tracer Lucifer yellow in LNCaP and 22Rv1 cells treated with 200 μM ALO for 24 h. **(D)** FITC-Dextran staining of LNCaP and 22Rv1 cells following 24 h of treatment with 200 μM ALO. Scale bar: 10 μm.

**Supplementary Figure S4. ALO induces lysosomal vacuolation. (A)** Immunofluorescence analysis showing the colocalization of Rab5 and LAMP1 in LNCaP and 22Rv1 cells treated with 200 μM ALO for 24 h. **(B)** Colocalization analysis of Rab7 and LAMP1 in ALO-treated prostate cancer cells. Scale bar: 10 μm.

**Supplementary Figure S5. V-ATPase is required for ALO-induced lysosomal vacuolization and cytotoxicity in prostate cancer cells. (A)** Bright field and **(B)** LAMP1 immunofluorescence images of LNCaP and 22Rv1 cells treated with 200 μM ALO, 1 μM BafA1, or their combination for 24 h, demonstrating that BafA1 rescues ALO-induced vacuolation. **(C)** Cell viability after as described in **(A)** for 24 h (n=3), determined via a CCK-8 assay, showing that BafA1 attenuates ALO-induced cell death. **(D)** qPCR validation of ATP6V1A knockdown efficiency in LNCaP and 22Rv1 cells (n=3). **(E)** Bright field microscopy images of ATP6V1A-knockdown LNCaP and 22Rv1 cells treated with 200 μM ALO for 24 h, demonstrating the essential role of V-ATPase in ALO-induced vacuolation. **(F)** Time-dependent viability of ATP6V1A-knockdown LNCaP and 22Rv1 cells treated with ALO, as assessed by a CCK-8 assay at 24, 48, and 72 h (n=3). **(G)** Images of LAMP1 immunofluorescence in ATP6V1A-knockdown LNCaP and 22Rv1 cells treated with 200 μM ALO for 24 h, revealing attenuated vacuolation. Data are presented as mean ± SD. *p < 0.05, **p < 0.01, ***p < 0.001. Scale bar: 10 μm.

 **Supplementary Figure S6.** **ALO alters the localization and distribution of V-ATPase subunits on the lysosomal membrane.** **(A-C)** The colocalization fraction between LAMP1 and ATP6V1A **(A)**, LAMP1 and ATP6V0D1 **(B)**, and ATP6V1A and ATP6V0D1 **(C)** upon ALO treatment. **(D)** Lysosomal pH was determined after 22Rv1 or RWPE-1 cells were stained with LysoPrime Green and pHLysRed, followed by incubation in each specified pH titration buffer with 10 μM nigericin and monensin (n=3). The lysosomal pH values of 22Rv1 or RWPE-1 cells were calculated based on the standard curve separately. **(E)** Representative immunofluorescence images and quantitative analysis of Galectin-3 puncta. 22Rv1 cells were treated with ALO over a 24-hour time course (0, 4, 8, 12, and 24 h). Cells were stained for Galectin-3 (green), LAMP1 (red), and nuclei (DAPI, blue). The bar chart displays the quantified number of Galectin-3 puncta per cell at each time point to evaluate lysosomal membrane permeabilization (LMP). Data are presented as mean ± SD. Scale bar: 10 μm.

 **Supplementary Figure S7. Effects of ion channel inhibitors and osmotic stress on ALO-induced cytotoxicity and morphological changes.** **(A)** Bright-field microscopy images of LNCaP and 22Rv1 cells treated for 24 h with 200 μM ALO alone or in combination with 100 μM EIPA, 200 μM NPPB, or hypertonic medium (1 mM sorbitol). **(B-D)** Viability of LNCaP and 22Rv1 cells treated with ALO alone or in combination with 100 μM EIPA (a Na+/H+ exchanger inhibitor), 200 μM NPPB (a Cl- channel blocker), hypertonic medium (1 mM sorbitol), or hypotonic medium for 24h **(B)**, 48 h **(C)** and 72 h **(D)** (n=3). Data are presented as mean ± SD. *p < 0.05, **p < 0.01, ***p < 0.001. Scale bar: 10 μm. **Supplementary Figure S8.** **Osmotic stress and ion channel inhibition attenuate ALO-induced lysosomal morphological changes. (A, B)** Immunofluorescence analysis of LAMP1 expression in LNCaP **(A)** and 22Rv1 **(B)** cells treated for 24 h with 200 μM ALO alone or in combination with 100 μM EIPA, 200 μM NPPB, or hypertonic medium (1 mM sorbitol). **(C, D)** Images of LAMP1 immunofluorescence in ATP6V1A-knockdown LNCaP **(C)** and 22Rv1 **(D)** cells exposed to hypotonic medium (1:4 dilution of complete growth medium in water) for 24 h. Scale bar: 10 μm. **Supplementary Figure S9.** **HSPA8 is a direct binding target of ALO. (A–D)** Representative MS/MS spectra identifying the HSPA8-derived peptide in LNCaP and 22Rv1 cells treated with 200 μM or 400 μM ALO, confirming that HSPA8 is a direct binding target. **(E)** Molecular docking analysis prediction of the binding mode between ALO and the yeast HSPA8 homolog. **Supplementary Figure S10. ALO exerts antitumor effects by targeting HSPA8.** **(A)** qPCR analysis of HSPA8 mRNA expression in LNCaP and 22Rv1 cells following 24 h of treatment with 200 μM ALO (n=3). **(B, C)** qPCR analysis verifying the knockdown efficiency of HSPA8 at the transcriptional level in LNCaP **(B)** and 22Rv1 **(C)** cells (n=3). **(D)** Immunoblot analysis confirming the downregulation of the HSPA8 protein in the corresponding knockdown cells. **(E, F)** Baseline viability of HSPA8-knockdown LNCaP **(E)** and 22Rv1 **(F)** cells under normal culture conditions (CCK-8 assay, n=3). **(G, H)** Viability of HSPA8-overexpressing LNCaP **(G)** and 22Rv1 **(H)** cells (CCK-8 assay, n=3). **(I, J)** In vivo tumor growth curves **(I)** and final tumor weights **(J)** of xenografts derived from WT or HSPA8-overexpressing (OE-HSPA8) 22Rv1 cells after ALO treatment (50 mg/kg, every 2 days). **(K, L)** Viability of HSPA8-knockdown LNCaP **(K)** and 22Rv1 **(L)** cells treated with 200 μM ALO (CCK-8 assay, n=3). Data are presented as mean ± SD. *p < 0.05, **p < 0.01, ***p < 0.001.

 **Supplementary Figure S11. HSPA8 plays a crucial role in ALO-induced lysosomal vacuolation. (A, B)** Images of LAMP1 immunofluorescence in HSPA8-overexpressing LNCaP **(A)** and 22Rv1 **(B)** cells treated with 200 μM ALO for 24 h, demonstrating attenuated lysosomal vacuolation. **(C)** Bright-field microscopy images of HSPA8-overexpressing LNCaP and 22Rv1 cells under the same ALO treatment conditions. **(D, E)** Representative MS/MS spectra of HSPA8-specific peptides immunoprecipitated from LNCaP **(D)** and 22Rv1 **(E)** cell lysates using an anti-HSPA8 antibody. **(F)** Immunofluorescence analysis and quantification of HSPA8 and ATP6V1A colocalization in RWPE-1 cells treated with 200 μM ALO or PBS for 24 h. **(G)** Immunoblot analysis of HSPA8 and ATP6V1A expression in HSPA8-knockdown LNCaP and 22Rv1 cells. **(H)** Analysis of HSPA8 expression levels in normal prostate tissues (n = 52) and prostate cancer tissues stratified by Gleason scores (Gleason 6, n = 44; Gleason 7-10, n = 454). **(I)** Kaplan-Meier curves for progression-free survival (PFS) in prostate cancer patients, stratified into HSPA8-Low (n = 329) and HSPA8-High (n = 169) expression groups. P-value was determined by the Log-rank test. **(J)** Scatter plot illustrating the positive correlation between HSPA8 and ATP6V1A mRNA expression levels in prostate cancer samples. Correlation coefficient = 0.684, P < 0.001. Data are presented as mean ± SD. *p < 0.05, **p < 0.01, ***p < 0.001.Scale bar: 10 μm.

 **Supplementary Figure S12**. **ALO attenuates HSPA8-mediated ATP6V1A degradation.** **(A)** Immunofluorescence analysis of ATP6V0D1 and ATP6V1A colocalization in 22Rv1 cells with HSPA8 knockdown or overexpression. **(B)** Colocalization analysis of HSPA8 and ATP6V1A in 22Rv1 cells with HSPA8 knockdown or overexpression. **(C, D)** qPCR analysis of ATP6V1A mRNA levels in HSPA8-knockdown LNCaP **(C)** and 22Rv1 **(D)** cells (n=3). **(E, F)** Immunoblot analysis of ATP6V1A protein expression in LNCaP **(E)** and 22Rv1 **(F)** cells subjected to nutrient starvation for 16 or 24 h. **(G, H)** qPCR validation of LAMP2A knockdown efficiency in LNCaP **(G)** and 22Rv1 **(H)** cells (n=3). **(I, J)** ATP6V1A mRNA levels in LNCaP **(I)** and 22Rv1 **(J)** cells treated with 200 μM ALO, under 24 h of starvation, and with 21 μM CQ alone or in combination (n=3). **(K, L)** Immunoblot analysis of LAMP2A and ATP6V1A protein expression in LAMP2A-knockdown LNCaP **(K)** and 22Rv1 **(L)** cells. **(M, N)** Representative images **(M)** and quantitative analysis **(N)** of the PA-mCherry1-KFERQ CMA reporter in LNCaP cells. The fluorescent puncta area per cell was evaluated after 16 h of treatment following photoactivation. **(O)** Surface representation of ATP6V1A with the KFERQ motif (^231^KEILQ^235^), highlighted in red, exposed for HSPA8 accessibility. Data are presented as mean ± SD. *p < 0.05, **p < 0.01, ***p < 0.001. Scale bar: 10 μm.

 **Supplementary Figure S13**. **ALO triggers lipid signaling. (A)** Heatmap visualization of differentially expressed genes involved in cholesterol biosynthesis pathways in 22Rv1 cells treated with or without ALO. **(B, C)** Viability of LNCaP and 22Rv1 cells treated with 200 μM ALO, 50 μg/mL water-soluble cholesterol, 1 μM AY9944, or their combination for 48 h **(B)** and 72 h **(C)**, as assessed by CCK-8 assays (n=3). **(D, E)** qPCR analysis of PDZD8 mRNA levels in PDZD8-knockdown LNCaP **(D)** and 22Rv1 **(E)** cells (n=3). **(F)** Validation of PDZD8 protein downregulation in knockdown cells by immunoblotting. **(G, H)** qPCR analysis of PI4K2A mRNA levels in PI4K2A-knockdown LNCaP **(G)** and 22Rv1 **(H)** cells (n=3). **(I)** Confirmation of the knockdown efficiency of PI4K2A by immunoblotting. **(J)** LAMP1 IF in PI4K2A-knockdown LNCaP cells treated with 200 μM ALO for 24 h. **(K)** Bright field images of PI4K2A-knockdown cells treated with ALO. **(L, M)** Viability of PDZD8-knockdown LNCaP **(L)** and 22Rv1 **(M)** cells treated with 200 μM ALO (CCK-8 assay, n=3). Data are presented as mean ± SD. *p < 0.05, **p < 0.01, ***p < 0.001. Scale bar: 10 μm.

**Supplementary Table S1. Primers were used in this study.**

| Target | Application | Sequence |
| --- | --- | --- |
| HSPA8 F; HSPA8 R; LAMP2A F; LAMP2A R; ATP6V1A F; ATP6V1A R; PDZD8 F; PDZD8 R; PI4K2A F; PI4K2A R; ATP6V1A-Q231A F; ATP6V1A-Q231A R; ATP6V1A-Q484A F; ATP6V1A-Q484A R; CMV-F; BGH-R | **qPCR**  **qPCR**  **qPCR**  **qPCR**  **qPCR**  **qPCR**  **qPCR**  **qPCR**  **qPCR**  **qPCR**  **PCR**  **PCR**  **PCR**  **PCR**  **plasmid sequencing**  **plasmid sequencing** | 5ʹ-ACCTACTCTTGTGTGGGTGTT-3ʹ; 5ʹ-GACATAGCTTGGAGTGGTTCG-3ʹ; 5ʹ-GAAGGAAGTGAACATCAGCATG-3′; 5ʹ-CTCGAGCTAAAATTGCTCATATCCAGC-3′; 5ʹ-GGGTGCAGCCATGTATGAG-3′; 5ʹ-TGCGAAGTACAGGATCTCCAA-3′; 5ʹ-GCTCATTGCTATTGGAGGTGTG-3´; 5ʹ-AGCTTTCTTCCAACTGGCCC-3´; 5ʹ-CGAGGCAATGACAACTGGCTGA-3ʹ; 5ʹ-GCCACCTTGATAACAGGCTCCT-3ʹ; 5'-TTGACTGGCgcaAGAGTCCTTGATGCCCTTTTTCC-3'; 5'-ACTCTtgcGCCAGTCAACAGAGGATGATTGGC-3'; 5'-GGAAATTCTGgcaGAAGAAGAAGACCTGGCAGAAATT-3'; 5'-CTTCtgcCAGAATTTCCTTAGCTTTCGTCCTC-3'; 5'-CGCAAATGGGCGGTAGGCGTG-3'; 5'-TAGAAGGCACAGTCGAGG-3' |

**Supplementary Table S2. siRNA and shRNA sequences used in this study.**

| Target | Application | Sequence |
| --- | --- | --- |
| NC; HSPA8; HSPA8; LAMP2A; LAMP2A ATP6V1A; ATP6V1A; PDZD8; PDZD8; PI4K2A; PI4K2A | **shRNA**  **shRNA #1**  **shRNA #2**  **shRNA #1**  **shRNA #2**  **siRNA #1**  **siRNA #2**  **shRNA #1**  **shRNA #2**  **shRNA #1**  **shRNA #2** | TCCTAAGGTTAAGTCGCCCTCG; GCAACTGTTGAAGATGAGAAA; GCTGGTCTCAATGTACTTAGA; CTGCAACCTGATTGATTA; GGCAGGAGTACTTATTCTAGT; GCAAUGGUUUGUUGAGAUAUU; CAAAGACCTTTGTCGGATA; GCTTAAAGTTACATTGCTAGA; CCGTCTTAAAGTTACGTTGTT; CCTCTTCCTGAGAACACTAAC; CCGTTCTCTCAGGAGATCAAA |
