# Supplementary figures and images for: Targeting the HSPA8‐CMA‐ATP6V1A Axis Triggers Lysosomal Hyperacidification and Catastrophic Vacuolation in Prostate Cancer
Source: Adv Sci (Weinh). 2026 Jun 19:e76165. Online ahead of print. doi: 10.1002/advs.76165 (PMC13336817; doi:10.1002/advs.76165)

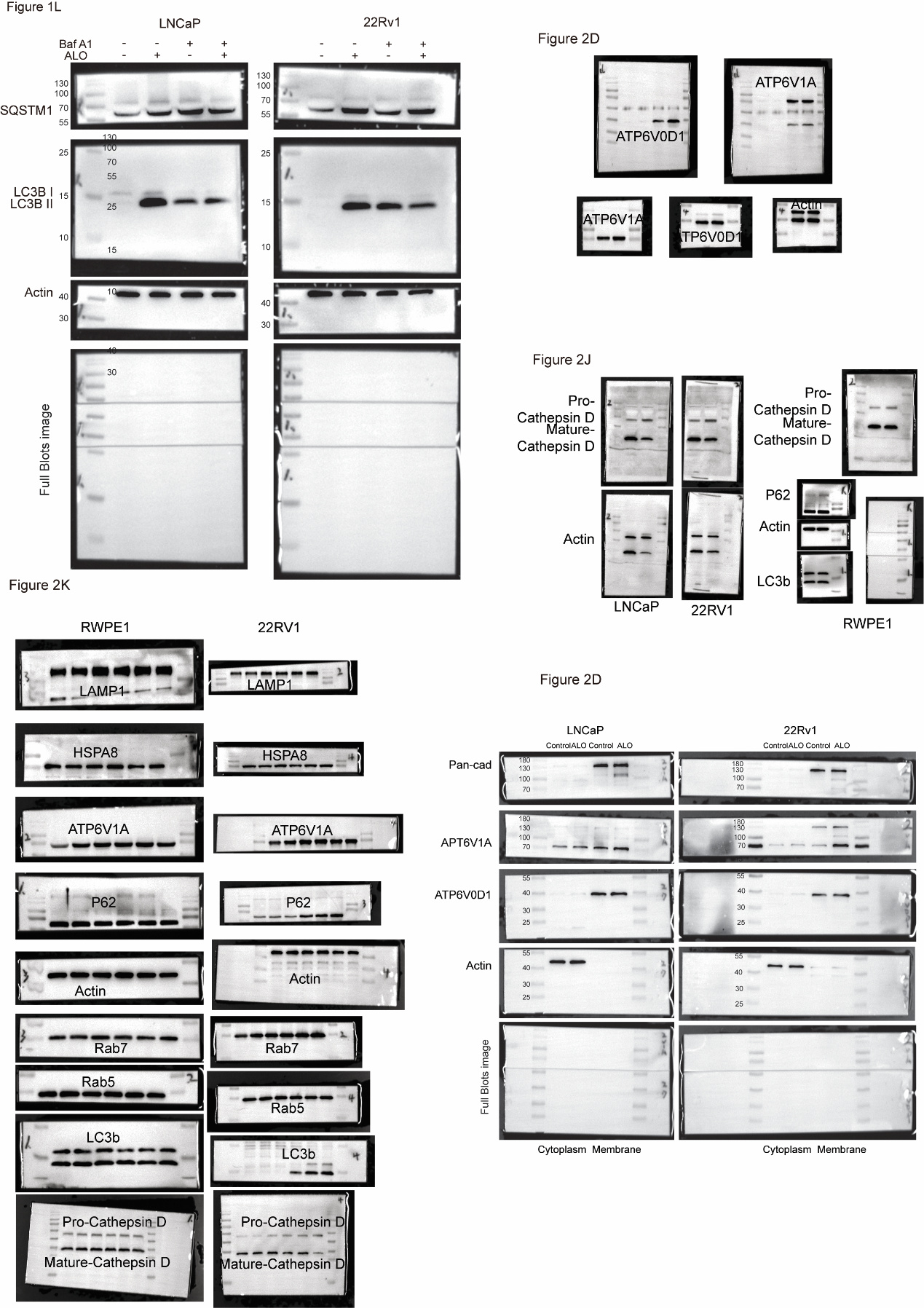

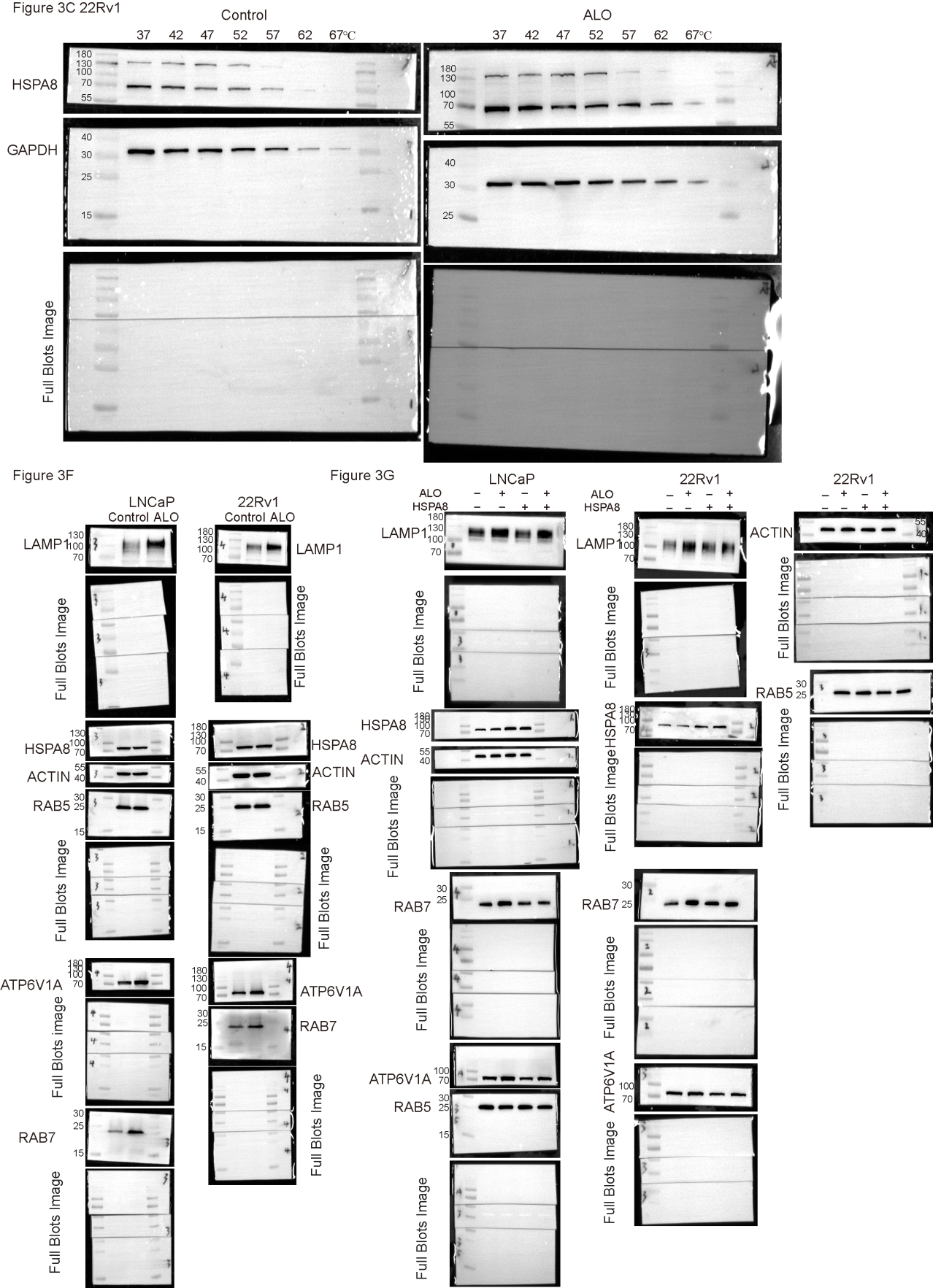

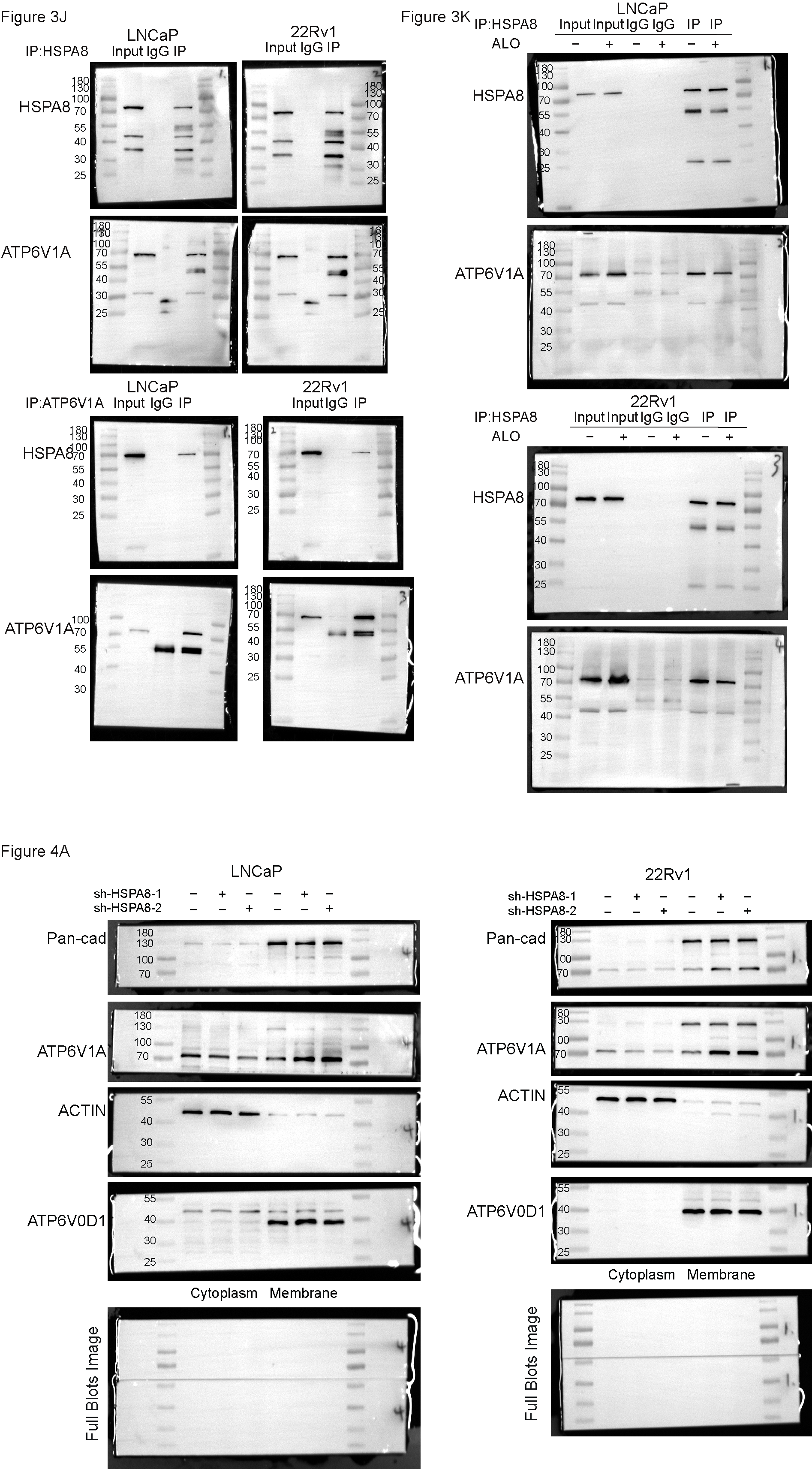

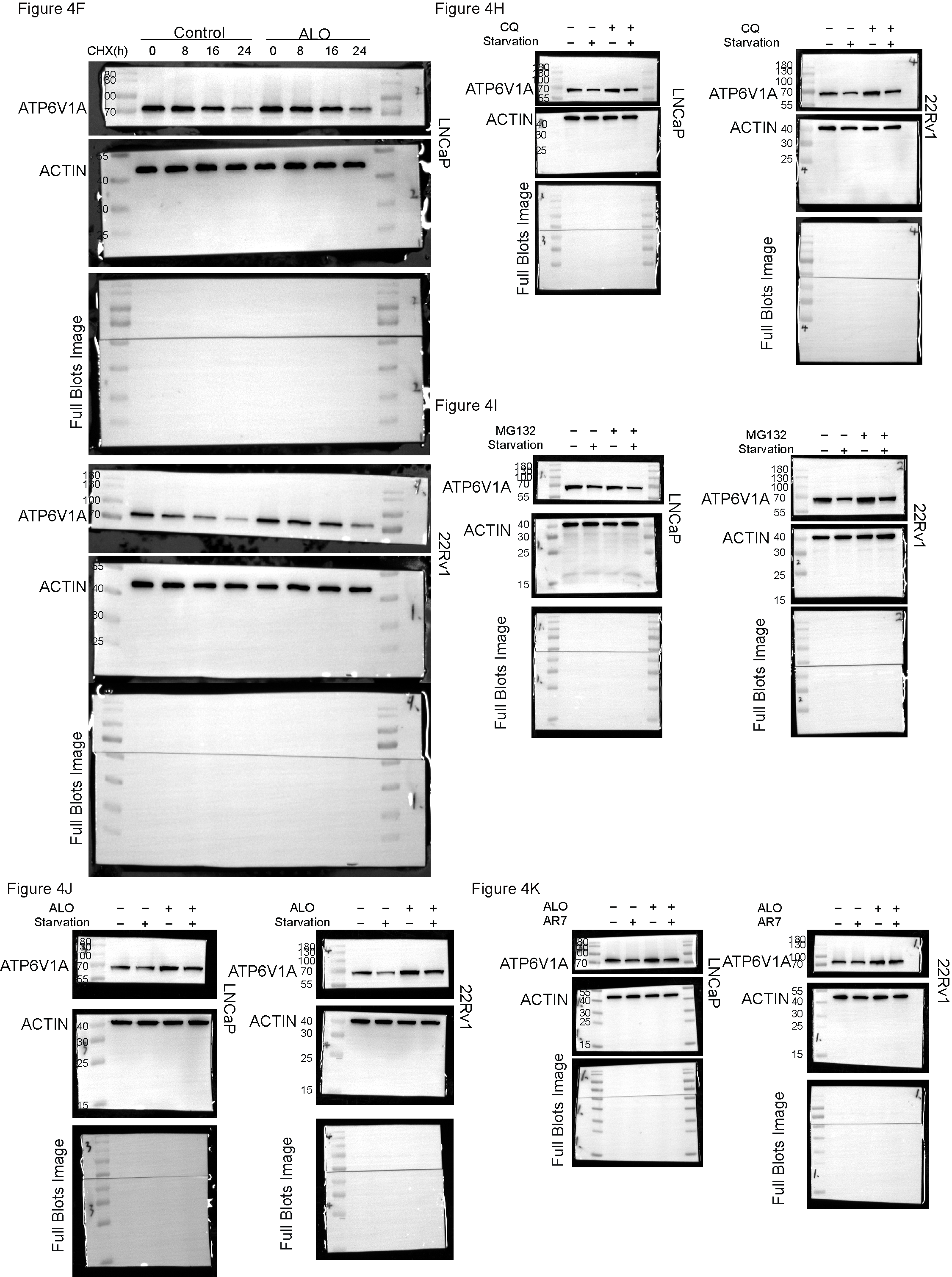

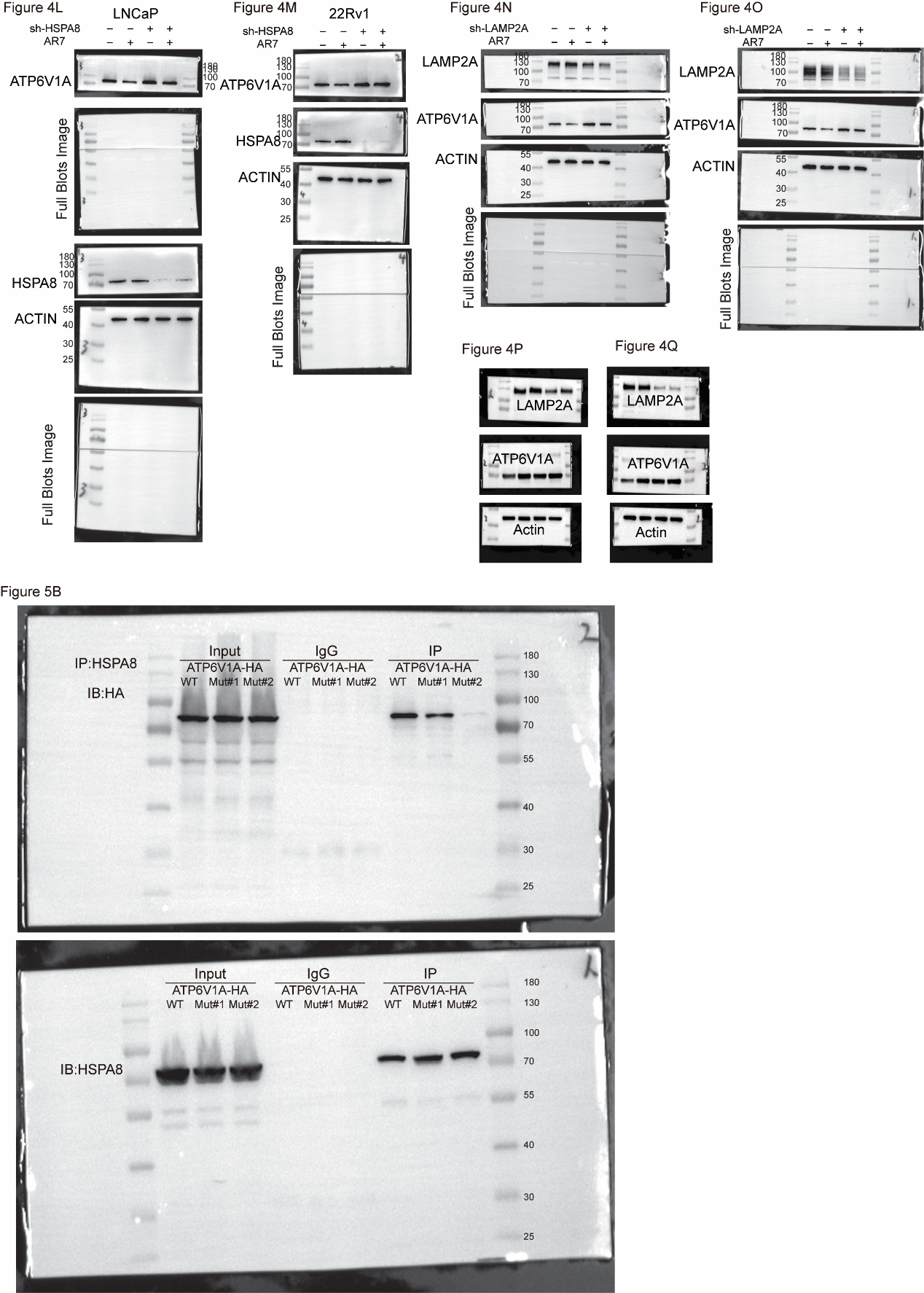

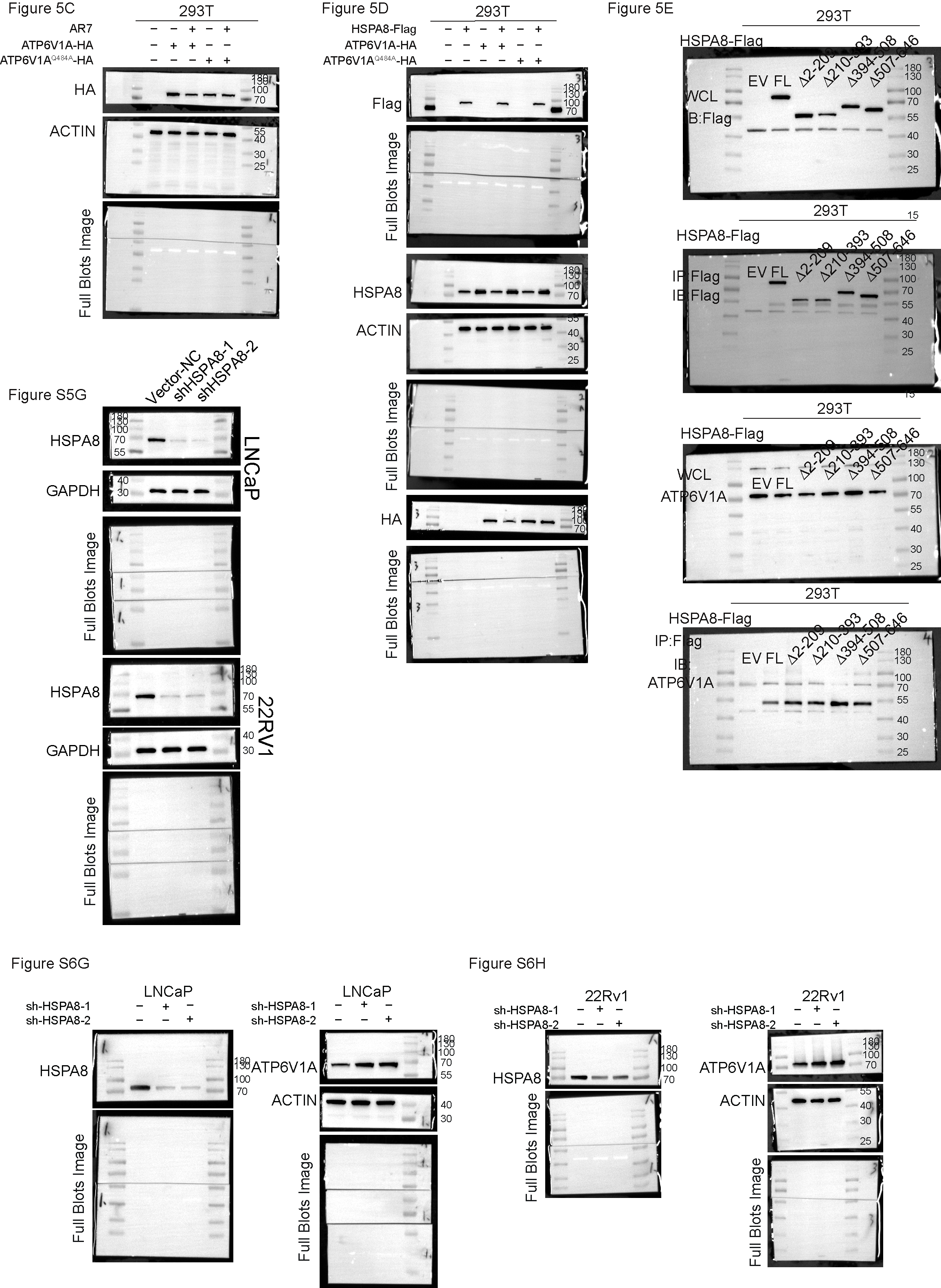

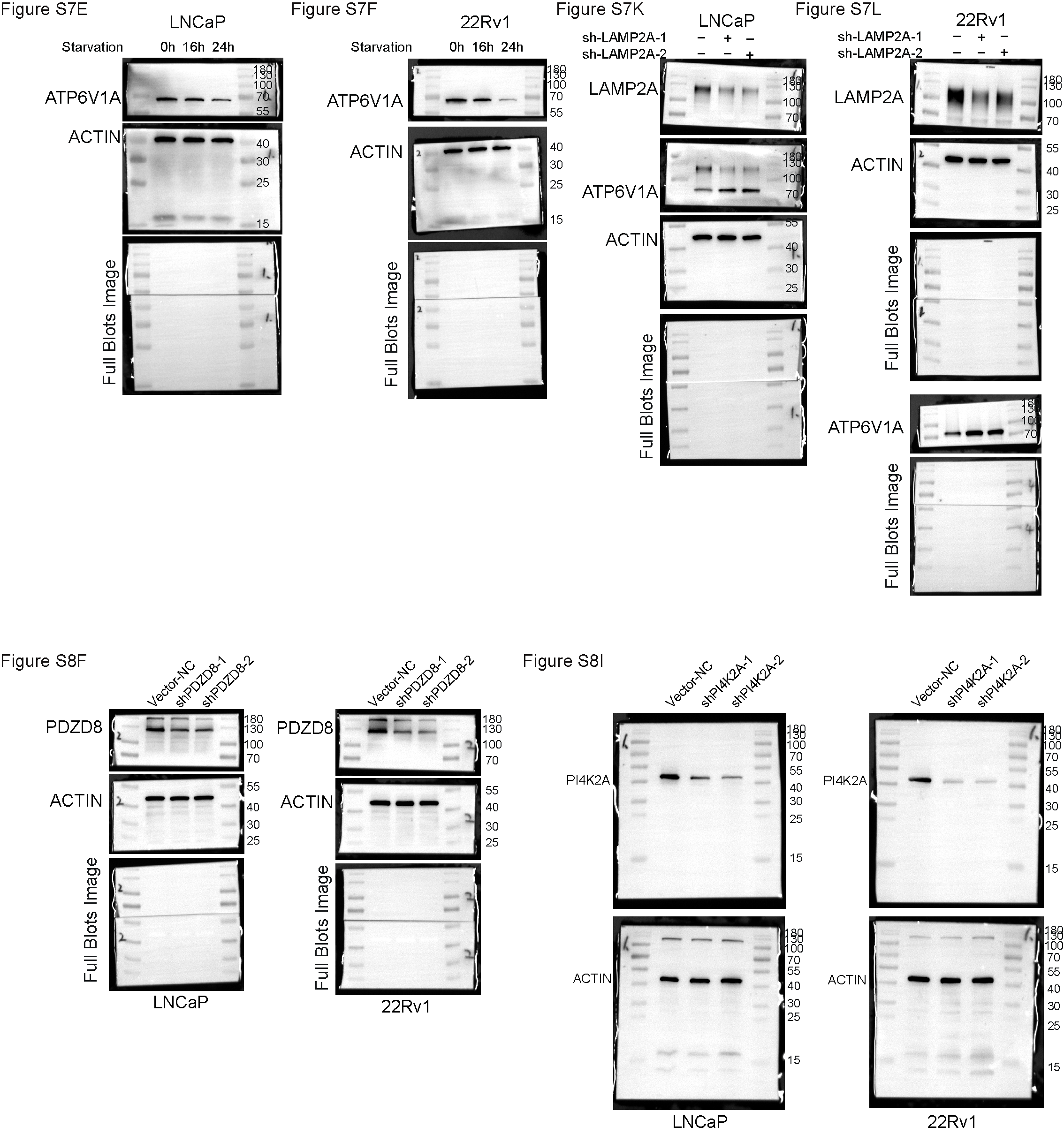

Supplement: Supplementary file 2 — Supporting File 2: advs76165‐sup‐0002‐SuppMat.docx. [file ADVS-9999-e76165-s001.docx]
